# Development and validation of a tool for detecting misinformation risk in diet, nutrition, and health content (Diet-MisRAT)

**DOI:** 10.1038/s41598-026-40534-2

**Published:** 2026-03-27

**Authors:** Alex Ruani, Michael J Reiss, Anastasia Z Kalea

**Affiliations:** 1 Curriculum, Pedagogy and Assessment, Institute of Education, University College London, London, WC1H 0AL UK; 2 The Health Sciences Academy, London, SW6 5UA UK; 3 Faculty of Medical Sciences, Division of Medicine, University College London, London, WC1E 6BT UK

**Keywords:** Misinformation risk assessment, Diet and nutrition misinformation detection, Public health communication, Infodemic management, Human-in-the-loop artificial intelligence, ChatGPT, Media literacy, Misinformation inoculation, Human behaviour, Psychology and behaviour, Risk factors, Health policy, Outcomes research

## Abstract

Misinformation in diet and nutrition is recognised as a major public health threat, with the potential to misguide dietary choices and contribute to preventable harm. To address this, we developed a Misinformation Risk Assessment Model (MisRAM), grounded in the World Health Organization’s hazard risk assessment principles. MisRAM conceptualises misleading content traits as stratifiable agents of informational adverse effects, weighed by their severity and likelihood of increasing recipient susceptibility. Building on this model, we designed the Diet-Nutrition Misinformation Risk Assessment Tool (Diet-MisRAT), a structured instrument that evaluates medium-to-long form content across four risk dimensions (inaccuracy, incompleteness, deceptiveness, health harm), yielding five-tier risk estimates from very low to very high. Validation involved five rounds: expert reviewers, trainee dietitians, postgraduate nutrition students, highly experienced nutrition professionals, and zero-shot prompt-based generative-AI risk detection. Results showed strong to very strong alignment with expert-derived benchmarks, supporting the tool’s interpretability and concurrent validity. ChatGPT demonstrated high test–retest reliability, accuracy, precision, sensitivity, and F1 scores under blinded untuned conditions, suggesting that adequately constructed, expert-designed prompting tools may help overcome training-dataset limitations. Diet-MisRAT offers a scalable, graded alternative to binary detection. Domain-calibrated risk stratification could guide proportionate interventions in content oversight, regulation, education, misinformation inoculation, and infodemic mitigation.

**Supplementary Information:**

The online version contains supplementary material available at 10.1038/s41598-026-40534-2.

## Introduction

Recognised as part of the World Health Organization’s (WHO) infodemic threat^1^, misinformation in diet and nutrition is a major public health concern, dangerously shaping dietary choices with potentially harmful consequences^2^. Exposure to misinformation can mislead health decisions, discourage essential care, and amplify preventable harm^3^. In the nutrition domain, frequent exposure to unfounded claims, ranging from extreme diets to unsafe supplementation marketed as curative, attests to the scale and persistence of this challenge^2,4,5^.

Herbal and dietary supplements alone have been estimated to account for 20% of drug-induced liver injury cases^6^ and approximately 23,000 U.S. emergency visits annually^7^. During the early COVID-19 pandemic, bleach-based ‘remedies’ gained traction online, coinciding with spikes in poison control calls and documented cases of severe toxicity from ingestible chlorine-dioxide blends dubbed Miracle Mineral Solution^8–11^. Around the same time, an international survey of over 3,700 individuals found that up to 43% had endorsed washing fresh produce with soap or bleach, a harmful practice promoted in a viral video^2^. Misinformation has also been implicated in decisions to abandon life-saving treatments, such as patients with curable cancers opting for unproven dietary alternatives, an approach linked to two-fold higher mortality rates^12^. Emerging clinical reports add to the evidence of misinformation’s dangers. One involved a man with alarming cholesterol-induced skin lesions from a carnivore diet^13^, a trend amplified among manosphere subcultures^14–16^. Another described hazardous metallic layering in a man’s colon from ingesting colloidal silver drops touted in some naturopathic circles^17^. A recent fatality involved an adolescent girl who died after adhering to a water-only fasting regime she reportedly discovered online^18^.

These tragic examples point to an urgent requirement for stricter safeguards to mitigate misinformation, particularly when health harm and the risk of death are at stake. Several countering approaches have been applied, including conventional fact-checking^19,20^, automated detection systems^21,22^, information quality appraisals^23,24^, crowdsourced platform moderation^25,26^, choice friction nudges^27,28^, media literacy education^29,30^, and psychological inoculation^31–33^. Each has influenced the broader infodemic response^34^, albeit variably. However, there remains a need to expand misinformation mitigation strategies in terms of scope, sensitivity, and scalability, particularly in addressing content that is not overtly false but may still pose a substantial risk of misleading recipients in ways that may lead to harmful outcomes^35^. A persistent challenge lies in the scarcity of content classification systems that move beyond reductive binary assessments^22,36,37^, and can instead capture gradations of misinformation risk. In the absence of mechanisms for stratifying such risk in content, digital platforms, practitioners, and regulators may be left without the means to prioritise responses or take proportionate action when faced with potentially harmful, though not categorically false, diet-health content.

Rather than a binary phenomenon, we conceptualise misinformation as a continuum of risk, much like exposure to a potentially harmful biological or chemical agent, where the degree of harm depends on the characteristics of the content, the context, and recipient susceptibility. It is not simply present or absent, but varies in severity and impact. Accordingly, instead of a dichotomous classification of content as either ‘misinformation vs non-misinformation’, ‘accurate vs inaccurate’, or ‘true vs false’, misinformation exists on a spectrum, with varying degrees of distortion, incompleteness, or misrepresentation. The risk of misinformation increases as content becomes more misleading or is more likely to be accepted as accurate, safe, or complete, despite its flaws and dangers.

Specifically in health contexts (including human and planetary health), we define ‘misinformation’ as the *extent* to which information might be perceived or accepted by a recipient as true, reliable, safe, or complete, despite it being demonstrably false, misleading, unsafe, or incomplete (whether accurate in parts or not), which can increase the risk of misunderstanding, poor decision-making, or harmful action. Importantly, the risk of health-related content misinforming a recipient is unlikely to ever be exactly zero, as a range of factors not only extrinsic but also intrinsic to the recipient may tip the scales towards greater susceptibility (e.g. cognitive capacity, intuitive thinking style, personal biases, literacy levels^38–40^). That said, when assessing misinformation risk, it is imperative to begin by examining the content itself, including its likelihood of misinforming recipients (particularly those who may be more susceptible^41,42^), based on empirical observations and predictive data.

For example, whilst a piece of online advice encountered by a recipient may be accurate, it could mislead or misinform by critical omission, such as not mentioning potential adverse effects, contraindications, or key safety precautions, thereby increasing the risk of harmful decision-making. To illustrate, pro-carnivore misinformation can mislead by emphasising nutritionally accurate benefits of meat, while omitting or downplaying ecological and health-harm risks of overconsumption^43–46^. Similarly, content claiming that extreme fasting can lead to rapid weight loss rests on a kernel of truth that increases its persuasive appeal, but misinforms by omitting serious dangers, such as loss of consciousness^47,48^, cognitive impairment^49,50^, metabolic disruption^51,52^, severe micronutrient deficiencies^53,54^, bone and muscle wasting^55,56^, and a heightened risk of triggering or worsening disordered eating behaviours^57,58^. In such instances, misinformation operates not through outright falsehoods, but through a selective presentation that masks health risks, increasing the likelihood of harmful (and in some cases life-threatening) outcomes, particularly among vulnerable groups like adolescents^59–62^.

Our recipient-centred, risk-based definition specific to health contexts broadly aligns with the American Psychological Association (APA) Consensus Report’s framing of misinformation as “any information that is demonstrably false or misleading, regardless of its source or intention”^32,63^, while extending it to include potential recipient-level impacts and harms. By applying a stratified understanding of misinformation, we were able to design an instrument structured around observable misinformation risk characteristics and their potential to mislead recipients, including possible harmful outcomes.

Building on this conceptual foundation, the objective of this study was to develop and validate the Diet-Nutrition Misinformation Risk Assessment Tool (Diet-MisRAT) to systematically estimate the risk of misinformation in relevant lay content. Grounded in preventive health and behavioural exposure mitigation, we aimed to offer a stepwise approach reflecting WHO risk assessment principles to support the prioritisation of misinformation risk management and communication strategies at scale, with intended applications across education, professional practice, regulation, and public policy.

### Development of a *de novo* misinformation risk assessment model

To inform instrument construction, we formulated a *de novo* Misinformation Risk Assessment Model (MisRAM) that considers not only the intrinsic characteristics of risk factors within content, but also their potential to mislead recipients and lead to adverse consequences, including harm. For this, we drew a conceptual parallel with WHO’s logic for human health risk assessment, described as a process intended to estimate the risk to a given target system or population following exposure to a particular agent of concern, accounting for both the agent’s inherent characteristics and those of the exposed system or population^64^.

**Fig. 1:**
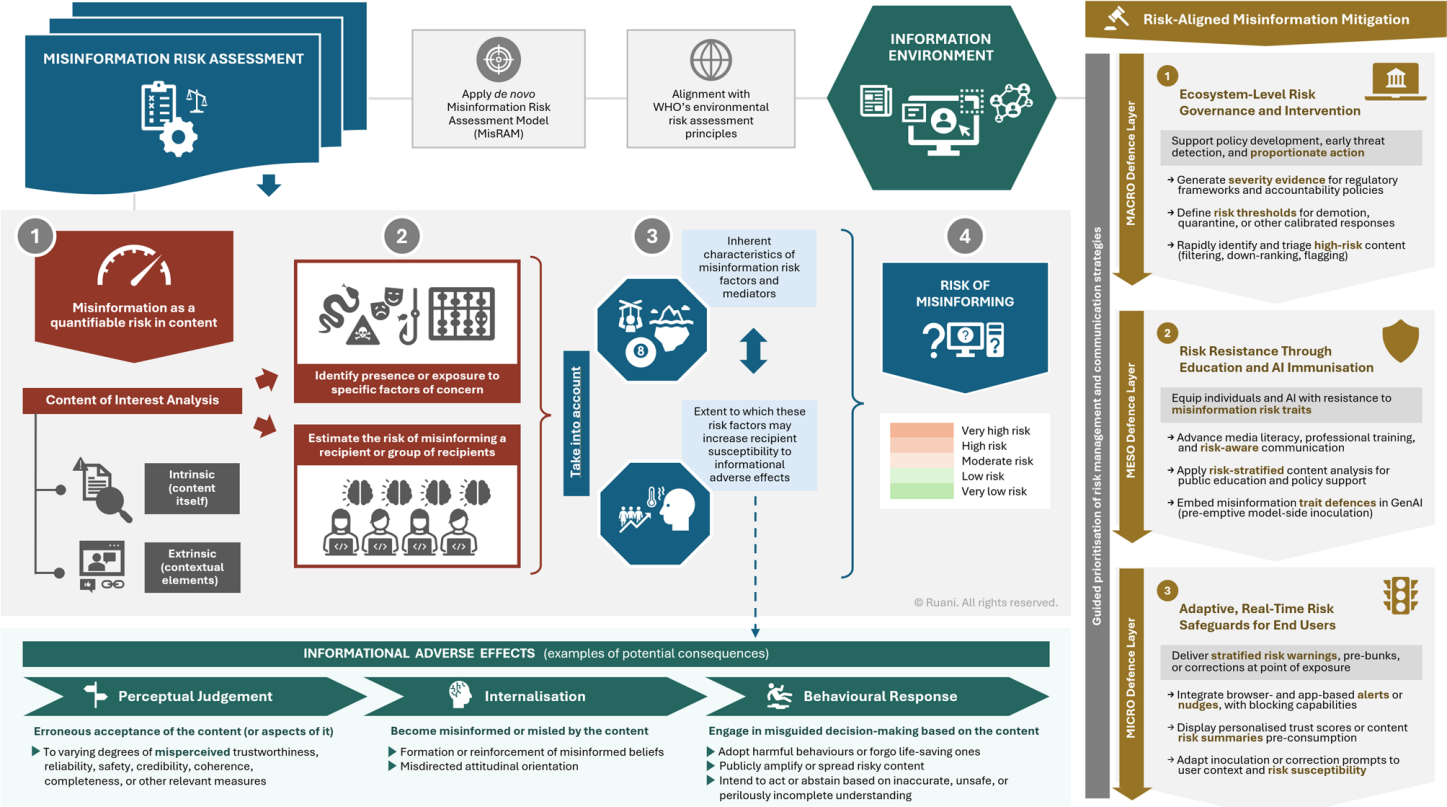
Susceptibility to informational adverse effects from misinformation exposure and risk-aligned mitigation. Flowchart delineates the sequential pathway through which presence or exposure to misinformation risk factors may lead to informational adverse effects, accounting for progressive stages of recipient susceptibility. The flowchart visually integrates the core stages of the Misinformation Risk Assessment Model (MisRAM) introduced in this work, beginning with the identification and evaluation of risk factors within a given information environment, susceptibility pathways, and eventual risk estimation. This framework thus treats misinformation as a quantifiable content-level risk. Content of interest can be assessed for the presence or exposure to specific factors of concern, taking into account their inherent characteristics and severity. Both intrinsic (within-content) and extrinsic (contextual) features can be considered in estimating the likelihood of misinforming recipients, progressing through three key susceptibility phases: perceptual judgement, internalisation, and behavioural response. The assessment would result in a stratified estimation of misinformation risk (very low to very high), offering a structured indication of the potential for informational harm. Such output can inform proportionate mitigation solutions, ranging from strategic prioritisation, education, and policy to platform-level oversight and tailored inoculation or correction prompts delivered via personal browsers, apps, and devices. To our knowledge, this represents the first formal adaptation of WHO’s environmental hazard risk assessment principles aimed at evaluating the threat of misinformation through structured content analysis, based on the presence and severity of content-level risk traits and recipient susceptibility.

Applying this logic to the informational landscape (Fig. 1), we defined ‘misinformation risk assessment’ as a process intended to estimate the risk of misinforming a recipient (or group of recipients) following the presence or exposure to specific factors of concern in content of interest, taking into account the inherent characteristics of these risk factors as well as the extent to which they may increase a recipient’s (or group of recipients’) susceptibility to:

- accept the content (or aspects of it) to varying degrees of *misperceived* trustworthiness, reliability, safety, credibility, coherence, completeness, or other relevant measures,
- become misinformed or misled by it (e.g. through the shaping or reinforcement of misinformed beliefs, or misdirected attitudinal orientation), or.
- engage in misguided decision-making based on it (e.g. adopting harmful behaviours or forgoing life-saving ones, publicly amplifying or spreading risky content, intending to act or abstain based on inaccurate, unsafe, or perilously incomplete understanding), among other informational adverse effects.

The process for misinformation risk assessment, formalised in the MisRAM model, consists of five practical steps guiding the extraction, classification, and stratification of misinformation risk factors within content of interest, forming the basis for tool development. This provides the foundation for tool-based estimation of misinformation risk on a graded scale: very low, low, moderate, high, and very high. These five developmental steps are detailed in the Methods section.

## Study phases and validation rounds

**Fig. 2:**
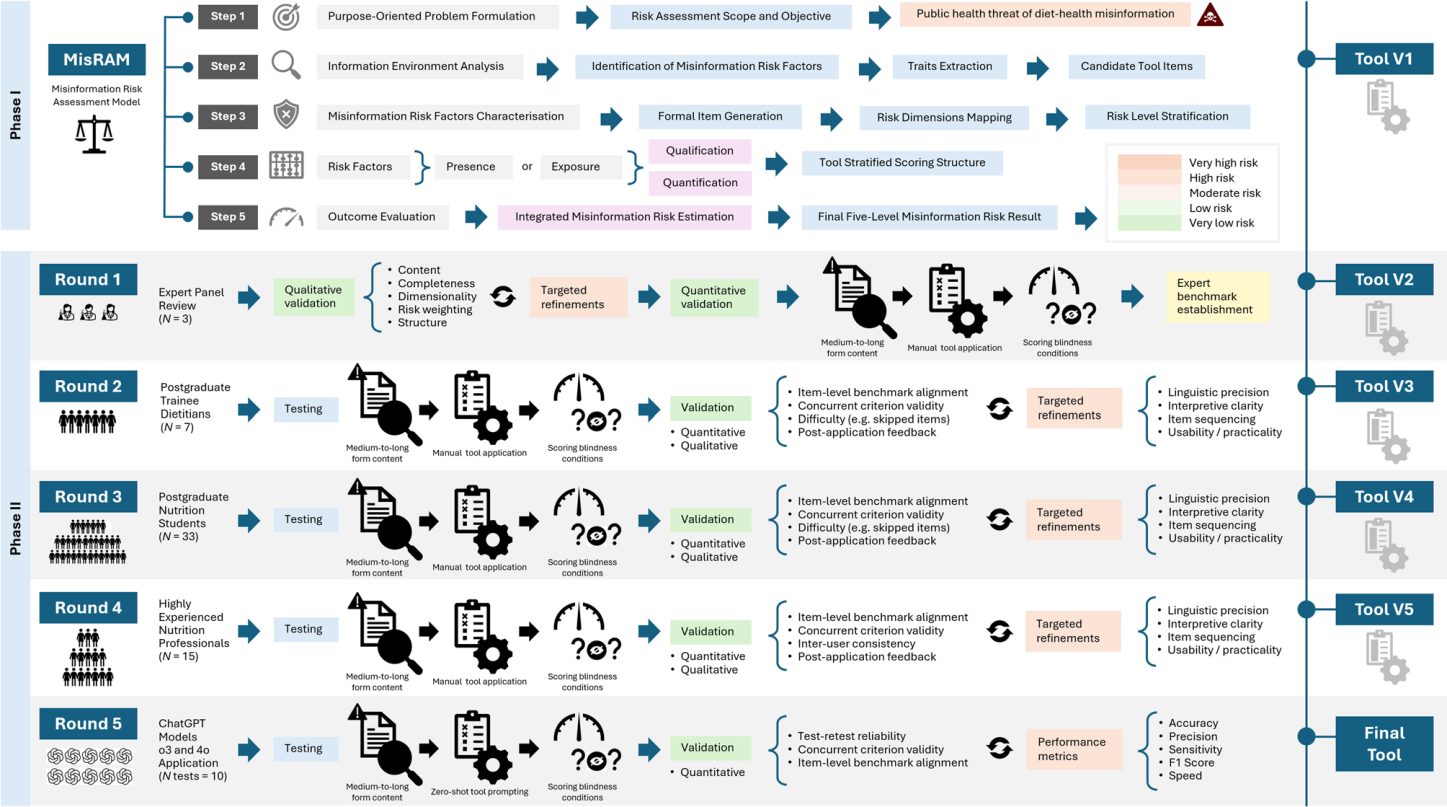
Study overview: two study phases and five validation rounds. Overview of the two-phase development and validation process for the Diet-Nutrition Misinformation Risk Assessment Tool (Diet-MisRAT). Phase I – Risk Assessment Modelling and Tool Development – involved the formulation of the Misinformation Risk Assessment Model (MisRAM), item generation, identification of four key risk dimensions (inaccuracy, incompleteness, deceptiveness, health harm), and stratified weighting for estimating misinformation risk in content. Phase II – Validation and Refinement – comprised five iterative testing rounds evaluating interpretability, alignment, and consistency through: (1) expert panel review and benchmark setting, (2) pilot testing with postgraduate dietitians in training, (3) testing with postgraduate nutrition students, (4) testing with highly experienced nutrition professionals, and (5) testing with ChatGPT (models o3 and 4o) under zero-shot prompt-based conditions. Each round contributed targeted refinements and validation outcomes towards the finalised tool.

To operationalise the study objective, we structured the development and validation of the Diet-MisRAT™ into two primary phases (see Fig. 2 for a study overview). Phase I – Risk Assessment Modelling and Tool Development – involved the formulation and implementation of our guiding risk assessment model (MisRAM), the generation and refinement of candidate items, the identification of key misinformation risk dimensions (Supplementary Table S1), and the allocation of a stratified weighting system to enable risk estimation in content of interest. Phase II – Validation and Refinement – comprised five iterative testing rounds combining expert judgement, specialised participant cohorts, and generative artificial intelligence (GenAI) to evaluate interpretability and consistent application:

- Round 1: Expert Panel Review and Benchmark Setting,
- Round 2: Pilot Testing with Postgraduate Dietitians in Training,
- Round 3: Testing with Postgraduate Students in Nutrition Science,
- Round 4: Testing with Highly Experienced Nutrition Professionals, and
- Round 5: Testing with ChatGPT Under Zero-Shot Prompt-Based Conditions.

Both phases contributed to the progressive refinement of the tool’s structure, clarity, and scoring logic, supporting the development of a final version that can systematically and reliably assess diet- and nutrition-related misinformation risk in medium- to long-form lay content.

## Results

### Phase I. Risk-Based modelling integration into structured tool design

The MisRAM process helped us to identify recurring traits and indicators of misinformation and classify them into interconnected risk profiles. We analysed both historical and contemporary content environments to extract and distil types of misinformation traits (e.g. inaccuracy cues, hazardous omissions, transparency issues, manipulative framing), exposure routes (e.g. reading, listening, watching), exposure characteristics (e.g. prominence, frequency), mediators (e.g. contextual amplifiers, sub-source precursors), and potential informational adverse effects (e.g. adoption of harmful behaviours, delay in seeking appropriate care). By isolating these features and linking them to observable or inferred recipient outcomes, we were able to formalise the main risk profiles of misinformation in a way analogous to pathogenic or toxicological risk assessments, where specific content traits act like agents of concern, exposure determines the magnitude of potential impact, mediators weaken cognitive safeguards or introduce latent vulnerabilities, and inherent trait characteristics and recipient susceptibility modulate the likelihood and severity of informational harm.

The first iteration of the Diet-MisRAT operationalised this model by evaluating both content-level risks and their possible recipient-level consequences. The result was a structured method for qualifying and quantifying misinformation risk in lay content related to diet, nutrition, and health, with outcomes stratified across five levels (from very low to very high) aimed to support tailored response guidance (see Supplementary Table S2 for tiered risk management and communication examples). For a description of the MisRAM and its five steps, see Methods.

### Phase II. Tool validation and refinement through iterative testing

Across all five rounds of testing, namely expert panel validation, two postgraduate student cohorts, a group of highly experienced professionals, and zero-shot AI model applications, the Diet-MisRAT demonstrated strong benchmark alignment, interpretability, and consistency.

#### Round 1: expert panel tool application and benchmark setting

Pearson’s correlation coefficients showed very strong alignment between each expert validator’s (second and third author) weighted responses and the tool developer’s (first author) preliminary benchmark (*r* = 0.94 and *r* = 0.97, both *p* < 0.00001; Supplementary Table S3). Subsequent item-by-item categorical comparisons reconfirmed high response-level agreement between the tool developer and the expert validators (Supplementary Fig. S2, panels a-c). Of all items, only one showed complete disagreement, which led us to update the benchmark response based on validators’ consensus. All remaining benchmark responses were retained without requiring revision. This outcome confirmed the consistency of item interpretation, response mapping, and scoring logic. The output was a validated reference dataset for subsequent human and AI testing rounds.

#### Round 2: pilot tool application with postgraduate dietitians in training

**Fig. 3:**
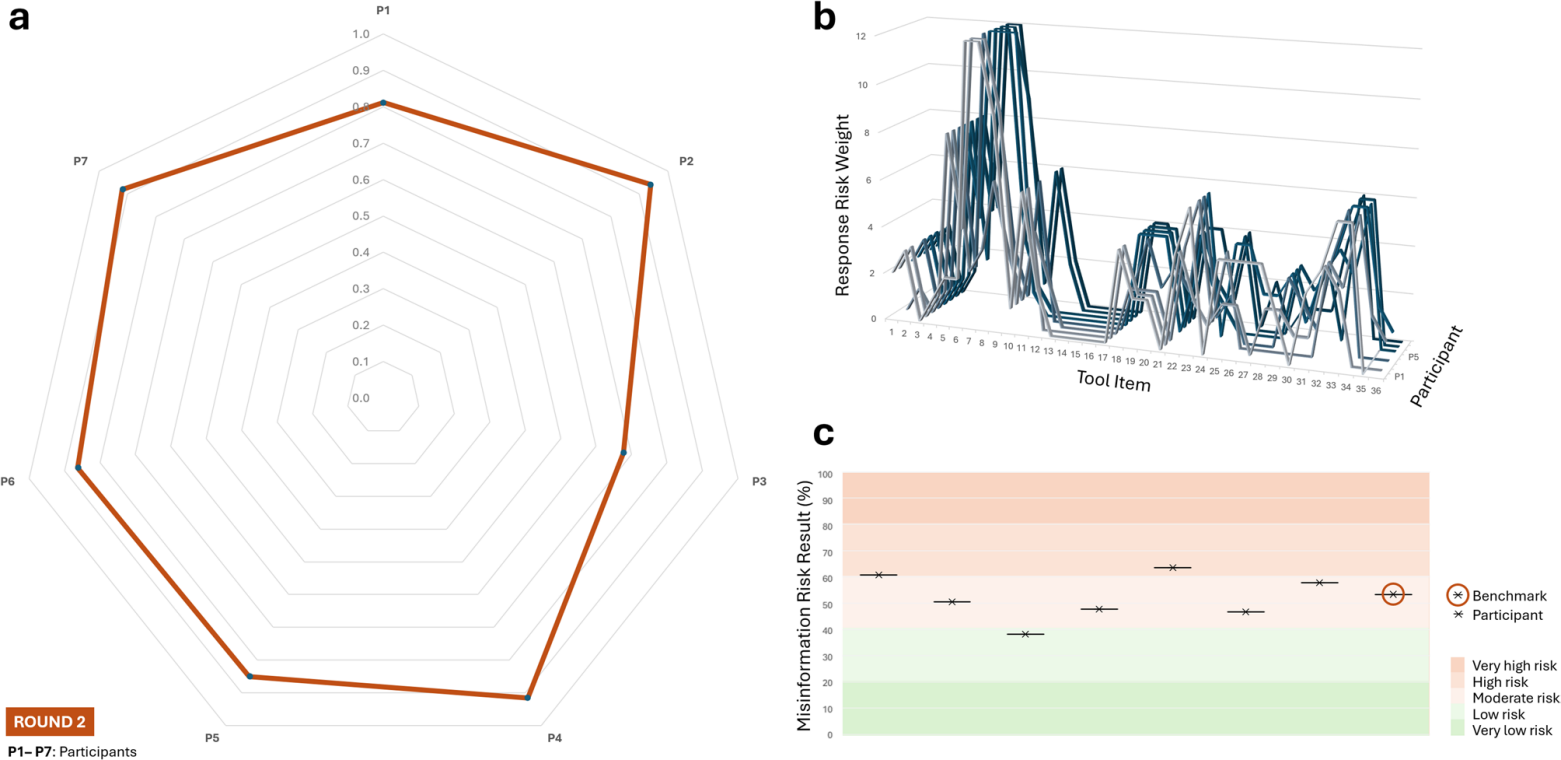
Round 2 concurrent validity, item-level response trends, and risk classification via the Diet-MisRAT. (**a**) Radar plot showing the strength of association between each participant’s item-level responses and the expert benchmark responses using Pearson’s correlation coefficient (*r*). All two-tailed comparisons were statistically significant (*p* < 0.00001). (**b**) Line graph comparing item-level scoring trends (response risk weights) across all tool items for each participant. Each line represents an individual participant’s scoring pattern. (**c**) Risk band plot with stratified background, displaying misinformation risk outcomes derived from each participant’s total tool score (black cross), expressed as a percentage of the maximum possible score after recalibration to a 100% scale. Background shading indicates five misinformation risk bands: very low (0–20.9%), low (21.0–40.9%), moderate (41.0–60.9%), high (61.0–80.9%), and very high (81.0–100%). Sample size: *N* = 7 (Round 2 cohort of postgraduate dietitians in training).

Results showed that trainee dietitians (*N* = 7) were generally able to apply the Diet-MisRAT in a way that closely reflected expert-derived standards. The median Pearson’s correlation coefficient (*r* = 0.86) between participant responses and the expert benchmark responses established a priori in Round 1 denoted strong positive alignment. Individual *r* values ranged from strong (0.81 ≤ *r* < 0.86; 43%, *N* = 3) to very strong (0.92 ≤ *r* < 0.94; 43%, *N* = 3), with only one moderate coefficient (*r* = 0.68). All correlations were statistically significant (*p* < 0.00001; Supplementary Table S3). These values supported the concurrent criterion validity of the tool when used by postgraduate student practitioners and represented strong interpretability, even among those with developing skills. See Fig. 3 (panels a-c) for a summary of concurrent criterion validity, item response trends, and categorical misinformation risk outcomes in Round 2.

Analysis of Round 2 data was conducted immediately following data collection and focused on item-level performance, skipped responses, and open-ended oral feedback gathered in a post-test group conversation. Based on this, the phrasing of various item prompts and their accompanying guidance text were fine-tuned for enhanced clarity, without compromising the tool’s conceptual integrity or original scoring structure.

#### Round 3: tool application with postgraduate students in nutrition science

**Fig. 4:**
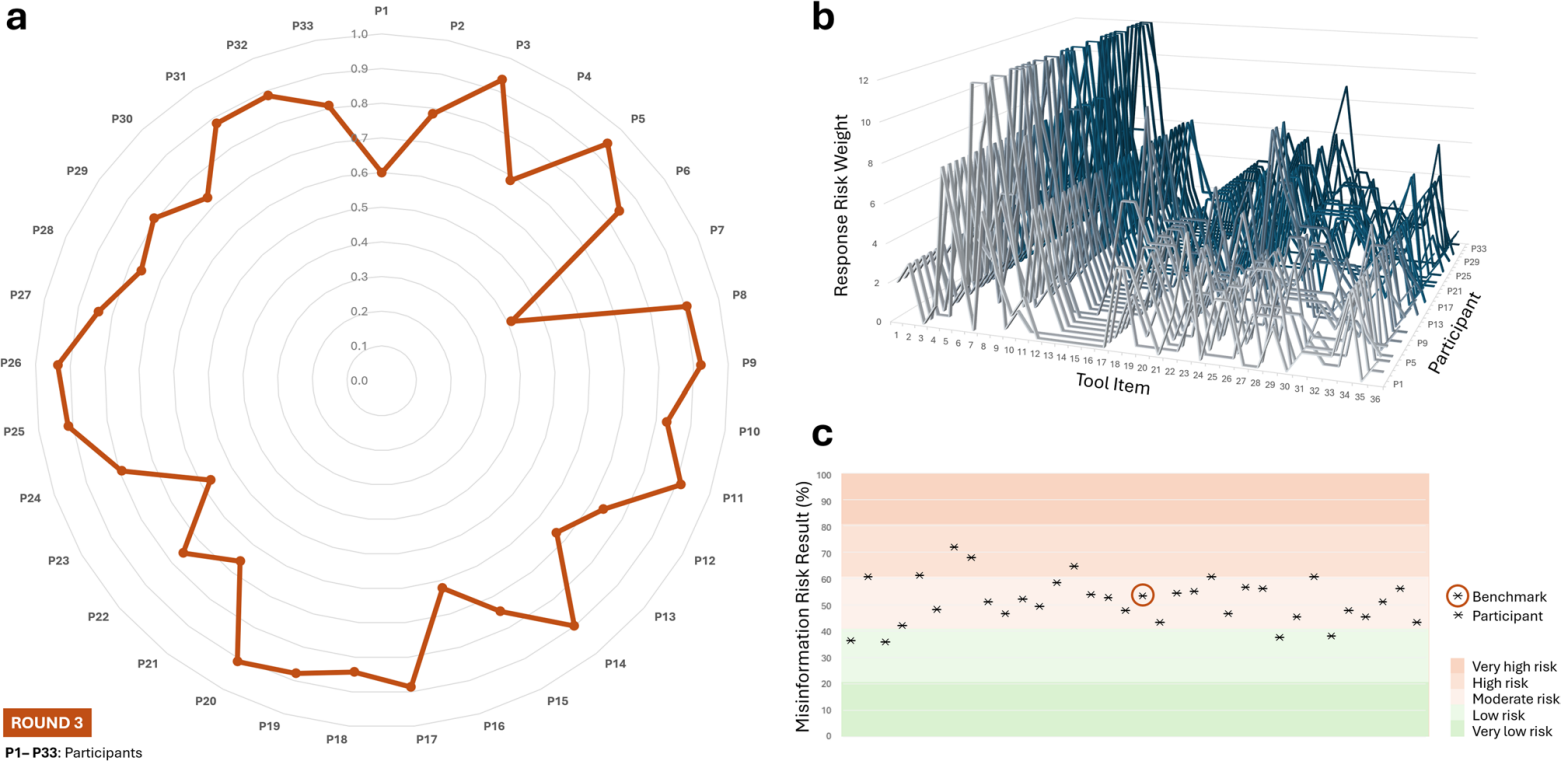
Round 3 concurrent validity, item-level response trends, and risk classification via the Diet-MisRAT. **(a)** Radar plot showing the strength of association between each participant’s item-level responses and the expert benchmark responses using Pearson’s correlation coefficient (*r*). All two-tailed comparisons were statistically significant (*p* < 0.012). **(b)** Line graph comparing item-level scoring trends (response risk weights) across all tool items for each participant. Each line represents an individual participant’s scoring pattern. **(c)** Risk band plot with stratified background, displaying misinformation risk outcomes derived from each participant’s total tool score (black cross), expressed as a percentage of the maximum possible score after recalibration to a 100% scale. Background shading indicates five misinformation risk bands: very low (0–20.9%), low (21.0–40.9%), moderate (41.0–60.9%), high (61.0–80.9%), and very high (81.0–100%). Sample size: *N* = 33 (Round 3 cohort of postgraduate nutrition students).

This third round demonstrated good concurrent criterion validity and supported the tool’s reliability as a structured risk evaluation instrument in a postgraduate academic setting. Most participants (79%, *N* = 26) in this round showed a strong (0.73 ≤ *r* < 0.89; 52%, *N* = 17) to very strong (0.91 ≤ *r* < 0.94; 27%, *N* = 9) positive correlation with the expert benchmark responses. Pearson’s *r* median (*r* = 0.83) reflected overall strong alignment, despite some outliers. All *p*-values (≤ 0.012) confirmed statistically significant correlations. See Fig. 4 for an overview of Round 3.

Oral feedback collected after using the tool indicated that respondents generally found it self-explanatory and appreciated its logical structure; however, a few identified specific items as more challenging to interpret. Insights and response data were reviewed and used to inform targeted refinements that aimed to enhance conceptual interpretability and linguistic precision, while preserving the tool’s overall structure and rigour in preparation for Round 4.

#### Round 4: tool application with highly experienced professionals

**Fig. 5:**
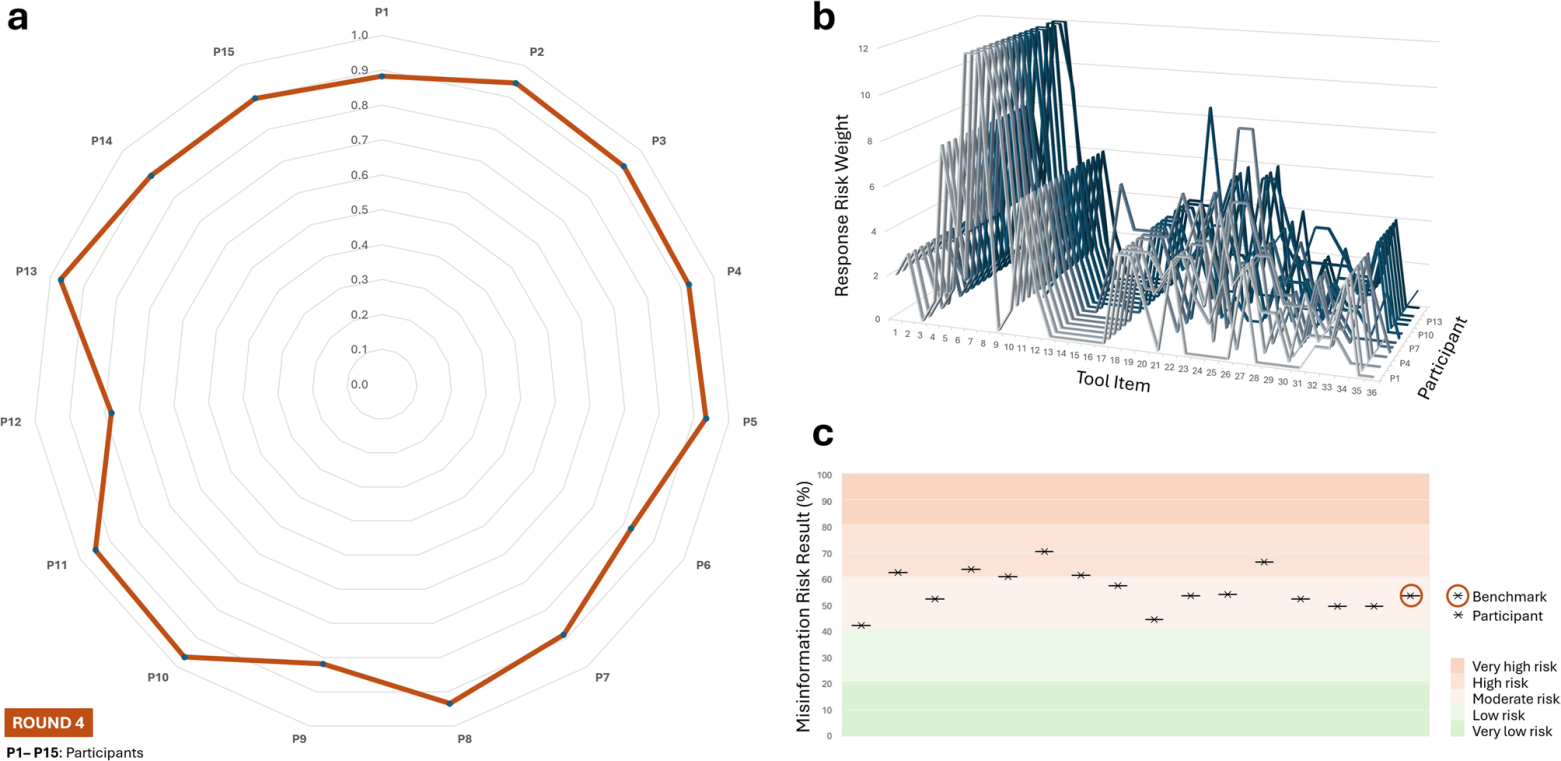
Round 4 concurrent validity, item-level response trends, and risk classification via the Diet-MisRAT. (**a**) Radar plot showing the strength of association between each participant’s item-level responses and the expert benchmark responses using Pearson’s correlation coefficient (*r*). All two-tailed comparisons were statistically significant (*p* < 0.00001). (**b**) Line graph comparing item-level scoring trends (response risk weights) across all tool items for each participant. Each line represents an individual participant’s scoring pattern. (**c**) Risk band plot with stratified background, displaying misinformation risk outcomes derived from each participant’s total tool score (black cross), expressed as a percentage of the maximum possible score after recalibration to a 100% scale. Background shading indicates five misinformation risk bands: very low (0–20.9%), low (21.0–40.9%), moderate (41.0–60.9%), high (61.0–80.9%), and very high (81.0–100%). Sample size: *N* = 15 (Round 4 cohort of highly experienced nutrition professionals).

This analysis involved 15 seasoned nutrition professionals, some with over three decades of experience, independently applying the Diet-MisRAT. All correlation coefficients were statistically significant (*p* < 0.00001), with a median Pearson’s (*r* = 0.92) reflective of a very strong alignment with the a priori expert benchmark. The majority of participants (60%) reached very strong correlation levels (0.90 ≤ *r* < 0.97), while the remainder (40%) fell within the strong correlation range (0.78 ≤ *r* < 0.89). Notably, no experienced professional fell below the strong threshold. See Fig. 5 for a summary of Round 4.

A Cronbach’s alpha coefficient (α = 0.73) observed in this round indicated acceptable inter-user consistency when the Diet-MisRAT was independently applied by experienced professionals. Because the tool assessed multiple interconnected misinformation risk dimensions and used a non-linear scoring system, both of which may attenuate observed inter-item correlations^65,66^, this value was interpreted as a reasonable lower-bound (i.e. minimum expected) level of consistency across participants.

#### Round 5: zero-shot prompt-based tool application by ChatGPT

**Fig. 6:**
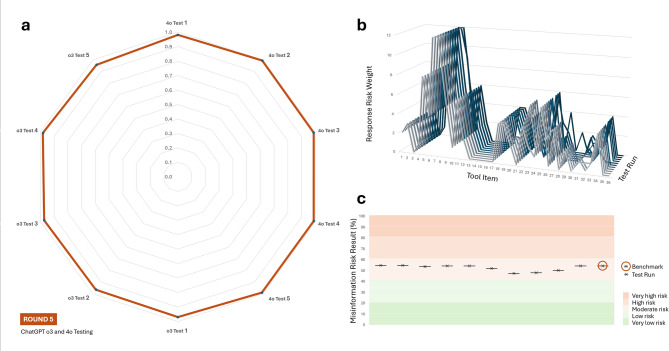
Round 5 ChatGPT test re-test reliability, zero-shot item-level response trends, and risk classification via the Diet-MisRAT. (**a**) Radar plot showing the strength of association between each ChatGPT test’s item-level responses and the expert benchmark responses, as measured using Pearson’s correlation coefficient (*r*). All two-tailed comparisons were statistically significant (*p* < 0.00001). (**b**) Line graph comparing item-level scoring trends (response risk weights) across all tool items for each ChatGPT test. Each line represents a distinct test instance conducted under zero-shot, untuned prompting conditions. (**c**) Risk band plot with stratified background, displaying misinformation risk outcomes derived from each test’s total tool score (black cross), recalibrated as a percentage of the maximum possible score. Background shading indicates five misinformation risk bands: very low (0–20.9%), low (21.0–40.9%), moderate (41.0–60.9%), high (61.0–80.9%), and very high (81.0–100%). Models tested in Round 5: ChatGPT 4o and o3 (*N* = 10 independent test runs under blinded-scoring in untuned, zero-shot conditions).

Round 5 of the Diet-MisRAT testing exhibited the highest alignment with the expert benchmark when compared to all human testing conducted in Rounds 2 through 4. Across all ten ChatGPT test runs, the median Pearson’s correlation coefficient (*r* = 0.98) demonstrated near-perfect concurrent criterion validity and test–retest reliability. Outputs from the ChatGPT 4o model yielded the strongest median correlation (*r =* 0.99) observed across both human and GenAI testing rounds. The ChatGPT o3 model also performed robustly, with a median Pearson’s (*r* = 0.97) indicative of very strong alignment, albeit marginally lower than the 4o model. Both model versions produced highly consistent results across repeated tool applications, with minimal variation between initial and follow-up outputs (all *p* < 0.00001). Figure 6 provides a summary ChatGPT’s test–retest reliability, item-level response trends, and misinformation risk outcomes (see Supplementary Table S4 for detailed test-by-test metrics).

ChatGPT’s 4o model demonstrated higher accuracy (mean 93.9%, SD ± 1.2, 95% CI: 90.4–97.4%) compared to the o3 model (mean 84.4%, SD ± 1.5, 95% CI: 79.1–89.7%) during all zero-shot applications of the tool. The narrower confidence interval for 4o denoted more consistent alignment with expert benchmark responses across test items, while the wider range for o3 showed marginally lower performance stability in interpreting and acting on the tool’s risk cues.

Precision levels were outstanding across test runs, with ChatGPT o3 achieving 100% in two instances (mean 98.1%, SD ± 1.7, 95% CI: 96.1–100.0%) and 4o maintaining consistent precision (mean 97.1%, SD ± 0.0, 95% CI: 94.7–99.6%). This indicated a strong ability to avoid overestimating risk (overflagging), even without any prior training or exposure to expert benchmarks, thus supporting the models’ aptitude for applying the tool’s decision prompts within the pre-established parameters.

Sensitivity was higher for 4o (mean 93.9%, SD ± 1.2, 95% CI: 90.4–97.4%) compared to o3 (mean 84.4%, SD ± 1.5, 95% CI: 79.1–89.7%) when following the tool’s instructions. In essence, 4o appeared more effective in applying the tool’s risk detection prompts and was less likely to underflag compared to the o3 model. This corresponded with item-level observations where o3 underestimated risk in a few instances.

**Fig. 7:**
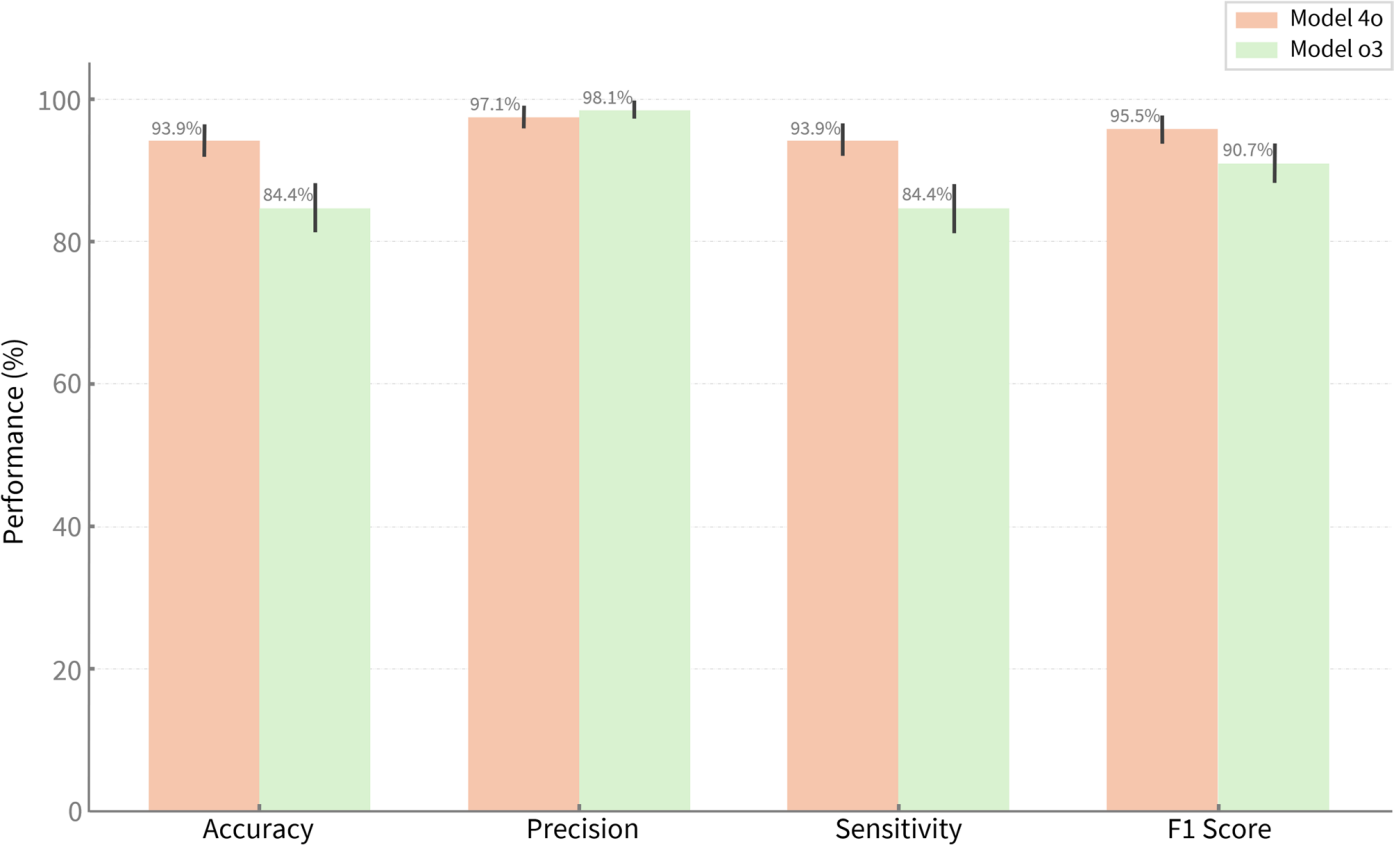
Performance metrics from Diet-MisRAT-guided ChatGPT zero-shot test runs. Bars represent mean performance (%) across accuracy, precision, sensitivity (recall), and F1 score, based on how each ChatGPT model (4o and o3) applied Diet-MisRAT items under blinded scoring in untuned, zero-shot, prompt-based conditions. Error bars show 95% confidence intervals.

The F1 score, representing the harmonic mean of precision and sensitivity, favoured model 4o (mean 95.5%, SD ± 0.7, 95% CI: 92.4–98.5%) over o3 (mean 90.7%, SD ± 0.7, 95% CI: 86.5–95.0%). This pointed to a more stable execution of the tool’s prompts by 4o, with fewer tendencies to overestimate or underestimate risk relative to the expert benchmark. Across all four performance metrics, the narrower confidence intervals observed for 4o denote greater stability in applying the tool’s items compared to o3. These results are summarised in Fig. 7, and detailed further in Supplementary Table S4.

Aggregated results from both models showed strong overall performance, with combined means of 89.2% (SD ± 5.1, 95% CI: 84.6, 93.7) for accuracy, 97.6% (SD ± 1.3, 95% CI: 95.4, 99.8) for precision, 89.2% (SD ± 5.1, 95% CI 84.6, 93.7) for sensitivity, and 93.1% (SD ± 2.6, 95% CI: 89.4, 96.8) for F1 score, each with narrow confidence intervals. Median values were closely aligned to respective means, reflective of performance stability across test runs. Model 4o showed higher medians across all four metrics (94.4%, 97.1%, 94.4%, 95.8%, respectively) than o3 (83.3%, 96.9%, 83.3%, 90.9%), which confirmed its slightly stronger and more consistent application of the tool’s risk assessment prompts (Supplementary Table S4).

## Discussion

To our knowledge, no existing approach has offered a structured content-level classification system that stratifies misinformation risk along a continuum. Most current detection methods have relied on binary labels (e.g. true/false, fake/real, verified/unverified)^21,22,67^, which restrict their utility in prioritising responses or tailoring mitigation strategies based on severity or potential for harm. In health and nutrition, the absence of verifiable falsehood does not equate to an absence of misinformational risk. True content that misleads by what it leaves out or how it manipulates perception, evokes misplaced trust, or conceals health-relevant precautions may even carry a greater risk of misinforming than inaccurate content that is noticeably false – precisely because it more easily escapes detection, appears credible, and is more likely to be accepted without scrutiny, while still shaping misinformed beliefs or harmful behaviours.

Roozenbeek and van der Linden^35^ similarly acknowledged the challenge of defining and intervening against misinformation that isn’t entirely false, a category that becomes especially problematic when content contains elements of truth but critical context is left out, leading to ambiguous veracity. As they noted, while verifiable falsehoods and uncontroversial facts pose fewer definitional or intervention hurdles, the real difficulties arise when dealing with ambiguous content that blends truth with dubious inferences. These cases can be difficult to remove, demote, or label, often escaping moderation or corrective measures due to the absence of outright falsehood. We sought to address this challenge through a risk-stratified approach to misinformation, which allows for the assessment of content not only on its factual accuracy but also on its potential to mislead or result in harm. By recognising that content need not be false to be informationally harmful, and that even accurate information can misinform via hazardous omissions, selective emphasis, decontextualisation, or deceptive framing, a graded risk model can enable more proportionate, adaptive, and layered interventions.

Our research presents the first misinformation risk assessment framework to formally establish a methodological parallel with WHO biological and toxicological hazard assessment principles for use in content analysis. Prior work in psychological inoculation theory has been instrumental in conceptualising misinformation as a contagious agent anchored in a virological analogy^33,68^, advancing our understanding of susceptibility and cognitive resistance. However, existing automated detection models^21,22^ and fact-checking tools^67,69^ remain incompatible with the foundational risk-grading logic underpinning hazard assessments – that is, an approach that evaluates risk not as a simple presence or absence (yes/no), but as a spectrum of severity based on exposure characteristics, agent properties, population susceptibility, and potential for harm. In contrast, MisRAM reconceptualises misinformation as a stratified risk, treating content as the medium and identifiable misleading traits as measurable risk agents known to increase recipient susceptibility. This reconceptualisation allowed us to design content assessment items based on empirically identified misinformation risk factors and graded risk estimates that mirror the logic of environmental hazard assessments. By doing so, we addressed a key methodological gap in evaluating diet-health content and its potential informational adverse effects, including risks to health.

The stratified risk levels calibrated through the Diet-MisRAT align with recent regulatory expectations, including Ofcom’s new risk assessment guidance for online platforms and search services concerning illegal and harmful content^70^. Although developed specifically for misinformation risk, the tool is consistent with Ofcom’s actionable approach to content risk severity, positioning it as a compatible solution within emerging digital governance frameworks. In line with these requirements, we devised the five-tier misinformation risk structure to facilitate targeted use for platform accountability, graded risk profiling, and safety evaluation (Supplementary Table S2**)**, particularly in contexts where proportionality and harm prevention are regulatory priorities.

To validate the Diet-MisRAT relevance, reliability, and usability, we sought input from professionals with domain-specific expertise. Experts in nutrition and education helped assess not only the adequacy of the tool’s items, but also their instructional clarity and interpretative precision. The iterative stages of Rounds 1 through 4 involved groups representing varying levels of subject-matter proficiency: senior academic expert validators, postgraduate dietitians in training, postgraduate nutrition students, and highly experienced nutrition professionals. Each round contributed to the quantitative evaluation of the tool’s practical application and reliability. Simultaneously, we used these rounds as qualitative refinement checkpoints to gather oral and written feedback, flag interpretative discrepancies, and iteratively strengthen the guidance text, item clarity and sequencing, and response cues. With each round, we progressively refined the tool for phrasing precision and design alignment with intended use.

Quantitative differences across validation rounds reflected variations in participants’ domain knowledge, educational background, and language proficiency. Most correlation coefficients in Round 2 indicated strong to very strong concurrent criterion validity, suggesting that even postgraduate dietitians still in training were generally able to apply the tool in a way that approximated expert expectations. While the tool demonstrated strong performance in Round 3, the overall alignment with the benchmark was slightly lower than in Round 2, likely influenced by participants’ more varied educational backgrounds (e.g. many without a prior undergraduate degree in nutrition) and lower English proficiency. These factors, combined with the conceptual demands of certain items and the need for precise interpretation of nutrition-related misinformation risk cues, may have contributed to the lower alignment observed in this cohort. As such, postgraduate nutrition programmes without a nutrition undergraduate prerequisite may benefit from incorporating formal training on identifying diet-health misinformation risk factors, especially those assessed by the Diet-MisRAT.

Given the tool’s requirement for both subject-matter expertise and the ability to interpret nuanced aspects of nutrition-related misinformation cues, we conducted Round 4 testing with highly experienced nutrition professionals to evaluate proficient tool application. The results from this skilled group exhibited the highest level of alignment with the benchmark criteria in all human rounds (second only to Round 1), reinforcing the tool’s suitability for professional use. Their ability to apply the Diet-MisRAT across complex, multidimensional items suggested that the instrument’s structure, prompts, and guidance were sufficiently clear. Most responses matched or near matched the expected risk allocations, without substantial deviations, indicating strong comprehensibility of the tool and a high degree of fidelity in its application.

Very minor interpretive differences across a few items did emerge, typically in those requiring more subtle distinctions, which we were readily able to address through additional clarification in the accompanying guidance text. Such subtle divergences were unsurprising and reflected the real-world variability in how even highly experienced professionals may perceive, prioritise, or weigh different risk markers^71^. Added to this, subjective judgments of misinformation risk, individual cognitive biases, domain familiarity, and interpretive differences among raters may influence scoring^72^. Nevertheless, the consistently high alignment between responses and the benchmark in this cohort provided strong evidence for the tool’s content validity and operational suitability under advanced expertise conditions. This round demonstrated that, when used by professionals with extensive domain knowledge, the Diet-MisRAT facilitated a replicable and reliable assessment of multi-dimensional misinformation risk. Research on larger samples could disentangle inter-rater variability and user-level bias by separating variance due to individual perception from variance due to tool structure or item ambiguity.

Round 5 testing using ChatGPT further supported the tool’s concurrent criterion validity. Models 4o and o3 applied the Diet-MisRAT with exceptionally high consistency relative to expert-derived responses, despite operating under fully blind-scoring and untuned conditions, with no prior supervised learning, no exposure to benchmark answers, and no human-in-the-loop feedback. Test–retest reliability remained very strong for both ChatGPT models, with stable application of the tool across independent test runs. Because large language models are subject to architectural updates, parameter changes, and inference drift over time^73^, future replication across model versions^74^ and different AI systems^75^ may help sustain confidence in the longitudinal robustness and reproducibility of Diet-MisRAT application.

Unlike classifier-based detection systems that depend on large pre-labelled datasets and extensive supervised training^22,76^, ChatGPT applied the Diet-MisRAT in a zero-shot manner, guided solely by the tool’s structured prompts and item-level instructions. Despite lacking exposure to benchmark outputs or training data input, both models achieved high accuracy, precision, recall, and F1 scores. These results suggest that, when equipped with well-designed, expert-validated prompts and item guidance, both general-purpose language models could reliably perform structured misinformation risk assessments with remarkably high fidelity. Provided that task instructions are appropriately constructed and aligned with domain-specific decision logic, our prompt-based, expert-derived approach may offer a scalable and interpretable alternative (or complement) to conventional machine learning detection systems. In particular, zero-shot prompting shows promise for overcoming the scarcity of adequately annotated training datasets in complex or specialised fields such as health and nutrition.

We acknowledge several limitations of this validation study. While we designed the tool with international applicability in mind, our testing centred on English-language content and participants largely trained in Western educational contexts. Future work should explore cross-linguistic and cross-cultural adaptability, particularly in regions with differing dietary norms, media environments, or public trust dynamics^42^. Though restricted in size, the participant pool was specialised and remained sufficiently engaged, notwithstanding the cognitively demanding and time-intensive nature of the tool application. Despite scoring blindness and high inter-rater alignment, interpretive variability persisted among users with weaker domain expertise or language proficiency, meriting tailored onboarding or decision-support overlays in broader rollouts. We also did not assess downstream behavioural impacts, such as whether tool use improved content judgement calibration or cognitive resistance against misinformation. That said, domain-relevant risk factors captured by the tool, particularly those tied to disinformation and health harm (Supplementary Table S2), hold promise for adaptation in psychological inoculation and media literacy interventions, especially among more vulnerable populations.

A methodological limitation is that we did not compute formal quantitative content validity indices (e.g. content validity index or ratio, kappa-based statistics). Diet-MisRAT was intentionally designed as a non-uniform, risk-weighted assessment instrument rather than a traditional psychometric scale, with items varying in number of response options, scoring ranges, and adaptive weighting (multiplier layers) according to risk severity. As a result, standard content validity coefficients, which assume item equivalence, uniform response structures, and equal contribution to a latent construct, were not straightforward to apply and risked yielding misleading estimates of item relevance or agreement. Instead, content validity was established through iterative expert review, benchmark refinement, and item-level consensus checks embedded within the validation rounds. Future work could explore adapted or model-specific content validity metrics better suited to non-uniformly weighted, multi-dimensional risk assessment tools.

Factor analysis was not conducted for the DietMisRAT, as the instrument was structured as a formative tool rather than a reflective scale. Each item in the tool was intentionally designed to represent one or more misinformation risk dimensions, rather than reflecting a single underlying latent construct. The tool includes items with heterogeneous response formats (ordinal with three to seven response options), each with expert-defined, non-monotonic scoring weights. These structural properties violate key assumptions required for factor analysis, including interchangeability of items, common scale metrics, monotonicity, and unidimensionality. We therefore did not apply conventional psychometric construct-validation procedures (e.g. exploratory or confirmatory factor analysis) or criterion comparisons with established scales. Consequently, high benchmark alignment (including in Round 5) should be interpreted as evidence of applied interpretability and concurrent agreement, rather than definitive evidence of theoretical construct validity. Subsequent research should extend validation by using approaches tailored to layered, adaptively weighted risk instruments, including generalisability-based reliability and convergent/discriminant comparisons against functionally analogous risk-assessment tools where available.

Sample sizes within each round were limited and cohorts were predominantly UK-based, limiting statistical power for advanced psychometric testing (e.g. multi-group comparisons) and restricting generalisability; however, this design intentionally prioritised depth of domain-expert feedback and iterative refinement under realistic use conditions. Broader testing across low-, moderate-, and high-risk materials (and varied formats and topics) is also needed to confirm stability of scoring and dimensional logic under more diverse real-world conditions.

Although the Diet-MisRAT is specific to the diet-health domain, the underlying MisRAM framework was developed to serve as a foundational model for constructing highly specialised misinformation risk assessment tools across other high-impact areas, such as medicine^77,78^, mental health^79,80^, sustainability^43,81^, consumer food choice^82,83^, food insecurity^84–86^, sociocultural phenomena (e.g. misogyny^87^), and beyond. Domain-specific instruments grounded in this logic could better account for unique content traits and misinformational harm pathways that general-purpose models often overlook^21,22^. Together, these innovations could facilitate more adaptive, context-sensitive risk detection and mitigation.

## Methods

### Phase I: risk assessment modelling and tool development

#### Formulation of a Five-Step Misinformation Risk Assessment Model (MisRAM)

To begin, we established a structured roadmap to characterise and assess risk factors in the informational environment, specifically those associated with misinformation. We did this by drawing on WHO’s stepwise methodology to risk assessment, originally devised for evaluating environmental hazards such as chemical and biological agents^64,88^. This roadmap, which we refer to as MisRAM (see Fig. 2), was designed to guide the structured development of tools capable of estimating the risk of misinforming recipients, based on the presence and nature of misinformation traits within any content of interest. In this study, we applied MisRAM specifically to diet, nutrition, and related health content.

The five-step MisRAM framework represented the underlying developmental logic to be used in Phase I to support tool construction. It guided the extraction, formalisation, and stratification of misinformation risk factors into a functional scoring instrument. These five standardised developmental steps, intended for use in any future tool built using MisRAM, are summarised as follows:

- Step 1. Purpose-Oriented Problem Formulation (*Risk Assessment Scope and Objective*).
- Step 2. Information Environment Analysis and Identification of Misinformation Risk Factors *(Traits Extraction and Candidate Tool Items)*.
- Step 3. Characterisation, Classification, and Stratification of Misinformation Risk Factors *(Formal Item Generation*,* Risk Dimensions Mapping*,* and Risk Level Stratification)*.
- Step 4. Risk Factor Presence or Exposure Qualification and Quantification *(Tool Stratified Scoring Structure)*.
- Step 5. Outcome Evaluation with Integrated Misinformation Risk Estimation *(Final Five-Level Misinformation Risk Result)*.

### Stepwise implementation of the MisRAM framework

The following five steps detail how the MisRAM framework was operationalised to construct the Diet-MisRAT tool.

#### Step 1. Risk Assessment Scope and Objective

We defined the scope and objectives of our risk assessment process, focusing on the public health threat posed by diet-health misinformation in medium- to long-form lay content, including headlines, subheadings, and other descriptive elements where present, as well as their relationship with the main material. Our goal was to create a scalable, multi-factorial, and stratified instrument^89^ capable of systematically estimating misinformation risk. We envisioned a structured prompting mechanism^90^ designed to be adaptable for both human and AI application, with potential for integration into automated and manual risk assessment workflows at scale^91^, ultimately enabling risk assessors, managers, and communicators (mainly relevant professionals, educators, researchers, and policymakers) to apply it alongside automated algorithmic systems for wide-scale impact.

#### Step 2. Traits Extraction and Candidate Tool Items

This step, comparable to hazard identification in environmental models, involved a methodical scan of historical and contemporary information environments, drawing from scientific literature, empirical analyses, and predictive modelling technologies. Salient patterns relevant to misinformation were identified and synthesised across sources through iterative review, comparison, and abstraction led by the tool developer. Data sources analysed included domain-relevant and psychosocial literature, multimedia content samples, case studies, case reports, and judicial records, enforcement and warning materials from regulatory and professional bodies (e.g. Advertising Standards Agency, British Dietetic Association), and annotated dataset insights (e.g.^92,93^), among others.

Following this scan and synthesis, we extracted recurring content traits, features, and markers by comparing repeated occurrences across sources and retaining those consistently associated with an increased likelihood of misleading recipients. Examples include variations of risky or hazardous omissions, framing distortions, decontextualisation, covert incoherence, emotionally manipulative tone, epistemic overreach, and radical opposition to established scientific consensus or life-saving medical approaches, among many other high-risk characteristics observed across sources (see Supplementary Table S1 for further examples). We also distilled additional aspects that may serve as ‘precursors’ to misinformation risk, including flawed or inexistent editorial process, undisclosed conflicts of interest, unrelated originator credentials (or lack thereof), reliance on unverifiable or subpar sources, and other mediating elements.

This step marked the initiation of item drafting, where each extracted risk factor was documented in a format conducive to future tool development. Through iterative cycles of content analysis and the extraction of recurring patterns consistently observed in high-risk materials, we generated a longlist of candidate items that reflected real-world misinformation risk traits and mechanisms observed across diverse information environments. The outcome was a practical repository of cues (the candidate list) for identifying misinformation risk factors in content of interest.

#### Step 3. Formal Item Generation, Risk Dimensions Mapping, and Risk Level Stratification

We formally generated tool items from the candidate list, classified them into interconnected misinformation risk dimensions (i.e. inaccuracy, incompleteness, deceptiveness, health harm; Supplementary Fig. S1 and Table S1), and applied a three-tiered risk stratification (low-, moderate-, high-risk items). Selected candidate items were converted into tool-ready prompts and formatted with clear supporting guidance notes and tailored response options reflecting real-world content characteristics. We sorted items based on their mode of action (e.g. manipulative framing, omission of adverse health effects) and intrinsic severity (e.g. potential to mislead or contribute to harmful choices). This informed which items (or combinations of content traits) warranted designation into a higher risk tier based on their mechanistic or cumulative impact. The aim was to prioritise the most consequential risk factors and reduce redundancy, refining the tool into a concise yet comprehensive structure.

#### Step 4. Tool Stratified Scoring Structure

Building upon the three-tiered risk stratification of items, we established the tool’s underlying scoring structure. Each response option within an item was assigned a base weight corresponding to the severity or risk profile of the misinformation factors it reflected. These weights were then multiplied by the item’s stratified risk level (low, moderate, high) as a coefficient to derive a final score per response option. This approach allowed for a severity-adjusted measurement of risk, a method seen in non-uniform scales weighing health content quality (e.g.^94^). By layering the scoring system, we were able to quantify both the density and severity of misinformation traits, and perform consistent comparisons across multiple content types.

#### Step 5. Final Five-Level Misinformation Risk Result

In this final leg, we sought to combine the outcome of the previous steps to help estimate the overall misinformation risk of content analysed. By summing the weighted item responses, the tool produced a total score that was then translated into one of five predefined risk categories: very low, low, moderate, high, or very high risk. This tiered risk result was designed to support actionable interpretations for stakeholders and automated systems (see Fig. 1 and Supplementary Table S2 for examples). Risk estimation outputs could help inform individual decision-making (e.g. whether to trust or share content), institutional responses (e.g. moderation, redirection, correction), and systemic applications (e.g. algorithmic risk flagging, platform-level warnings or demotion, gamified inoculation prompts in end-user browsers, devices, or interfaces). Our outcome-oriented logic mirrors the WHO’s final risk characterisation step, where hazard, exposure, and dose-response are combined into a concluding decision-supporting risk statement. The first tool iteration was thus completed to facilitate rapid, scalable misinformation risk estimation across a variety of content materials, grounded in established WHO risk assessment principles.

### Phase II: validation and refinement process

Following the development of the initial formal tool version, Phase II focused on validation and refinement through a series of structured testing rounds (Fig. 2).

#### Round 1: expert panel review, validation, and benchmark setting

To ensure content and construct validity, the first official version of the Diet-MisRAT underwent an in-depth review by an expert panel composed of two highly experienced professors with extensive research backgrounds and combined academic teaching experience exceeding 45 years. One expert brought specialised knowledge in nutrition science and dietetics, while the other contributed expertise in science education, pedagogy, and assessment, together offering an interdisciplinary perspective that informed the foundational validation and refinement process. The panel was not involved in initial tool development, only refinement of the drafted version.

The expert panel reviewed each tool item for its usefulness and essentiality in assessing misinformation risk, including its clarity, sequence, and conceptual fit. They independently assessed the completeness, phrasing, and ordering of the response options, each of which was structured with the highest-risk choice presented first to orient the user and anchor risk scaling. The panel provided oral and written feedback on the interpretability of user-guidance instructions and the clarity of item-specific language, particularly where answer options contained mixed or qualitative elements.

The tool’s overall structure was evaluated for logical progression, coherence across thematically related items, and the clarity of subsections. The scoring system was also assessed by verifying that higher-risk responses were appropriately weighted with greater penalty values, and that lower- or zero-risk responses reflected proportionately lesser or neutral scoring. The panel’s feedback informed the merging or elimination of items where overlap or redundancy was identified, as well as adjustments to guidance wording and scoring to enhance usability, comprehensiveness, and interpretive consistency across users.

#### A priori benchmark development

To establish a reference standard for the most appropriate response options when applying the Diet-MisRAT, we developed an a priori benchmark dataset that served as the comparative foundation for subsequent human and AI testing rounds. This process involved the independent application of the tool by the developer and both Round 1 expert validators, followed by a structured item-by-item agreement analysis.

##### Step 1. Developer and panel tool application process

The tool developer first applied the Diet-MisRAT to a variety of medium- to long-length lay content (including headlines, snippets, subheadings, main body text, and editorial elements such as author credentials) to explore the tool’s performance across different formats, framings, and topic types from both reputable and questionable (yet popular) sources. This initial testing informed the selection of a moderate-risk web article that exhibited a mix of misinformation traits with varying severity and prominence, and also reflected the complex nature of real-world content typically encountered by general audiences. The developer then generated a complete set of benchmark responses for the sample content, based on the intended interpretation of each item and its accompanying guidance. These responses reflected the developer’s conceptualisation of how tool items were meant to be applied, taking into account the specific intent behind each item and the subtle distinctions the tool made between different types of misinformation risk traits. This response set served as the preliminary benchmark.

To evaluate and refine this benchmark, the expert-panel validators independently applied the tool to the same sample content. Neither validator had access to the developer’s answers, nor to the scoring allocations for each item or the overall outcome of their assessment. Each used only the item prompts, guidance text, and response options provided in the tool, which was purposefully designed to be self-explanatory.

##### Step 2. Agreement analysis and benchmark refinement

Besides computing Pearson’s correlation coefficients to assess alignment between the tool developer and expert validators, we also conducted an item-level agreement analysis using categorical comparisons. Responses from the tool developer and expert validators were compared on a per-item basis, treating answer choices as unweighted categorical selections. Each item response was classified according to whether it matched or diverged from the developer’s original selection. These comparisons were intended to identify patterns of agreement or discrepancy and to guide potential adjustments to the benchmark before subsequent testing rounds.

Where diverging responses were observed, a majority-rule approach was applied to determine whether a revision to the benchmark was warranted. If both expert validators selected the same alternative response that differed from the developer’s original answer, the benchmark was updated to reflect the consensus choice. If at least one expert validator matched the developer’s answer, the original benchmark was retained. Aiming to minimise the influence of individual bias, this procedure ensured that each benchmark response was based on a convergent understanding of the item’s purpose and confirmed through expert consensus.

#### Round 2: pilot testing with postgraduate dietitians in training

Seven trainee dietitians based in the United Kingdom (see Appendix A for demographics) participated in a pre-test to evaluate the preliminary version of the Diet-MisRAT. Informed consent was obtained from all participants prior to the activity. The session was conducted in a meeting room within a hospital setting in March 2024, with all participants completing the task simultaneously over approximately 45 min. Each participant independently applied the tool to the same medium-to-long-form sample content, using either printed forms or a provided URL to access both the assessment questionnaire and the content to be analysed. Following the application, oral feedback was collected during a post-test group conversation, guided by questions on clarity, interpretation, and usability.

#### Round 3: testing with postgraduate students in nutrition science

The research was conducted in person in a classroom setting in March 2024, with postgraduate nutrition students in the United Kingdom voluntarily completing the activity simultaneously. Respondents were given approximately 45 min and provided with a URL to independently access the tool interface and sample content for analysis. One participant opted to complete a paper version instead. After completion, respondents were invited to provide open-ended oral feedback on the tool’s phrasing, interpretability, and usability.

A total of 50 respondents provided consent to participate in Round 3 of the study. Of these, 16 were excluded from analysis for abandoning the questionnaire partway through, and one additional respondent was removed due to unusually rapid completion (3 min and 6 s – indicative of random clicking). Consequently, the final sample size for analysis comprised 33 participants (see demographics in Appendix A).

#### Round 4: testing with highly experienced nutrition professionals

Since the tool demands both subject-matter familiarity and the capacity to interpret complex nutrition-related misinformation concepts, Round 4 involved testing with highly experienced nutrition professionals to evaluate the tool’s applicability, clarity, and interpretability under expert use. Fifteen senior nutrition professionals, each with extensive career experience ranging from around a decade (inclusion criterion) to over 30 years, took part in this round. Participants were based in the United Kingdom (except for one in the United States), and had recognised standing in research, higher education, science communication, clinical dietetics, and/or public health. The majority (87%, *N* = 13) held postgraduate qualifications at the master’s and/or doctorate levels (other demographics in Appendix A).

Participation was voluntary and conducted online independently at a time of each participant’s choosing during the collection period from April to June 2024. After providing informed consent, each participant used the tool to assess the same medium-to-long content sample, with an average completion time of 37 min, 36 s. Following this, both oral and written feedback (within the same questionnaire) were collected to capture expert reflections on usability and interpretive clarity. Responses were examined to assess the tool’s applicability in a professional context, and minor clarifications and refinements were made in light of this expert input.

#### Round 5: testing with ChatGPT under zero-shot prompt-based conditions

In this final testing round, we engaged the large language model (LLM) Generative Pretrained Transformer (GPT), developed by OpenAI – one of the most widely used multimodal generative artificial intelligence (GenAI) end-user interfaces available at the time of this research. We instructed ChatGPT’s model 4o (labelled ‘great for most questions’) and subsequently model o3 (labelled ‘uses advanced reasoning’; Appendix B) to apply the Diet-MisRAT to the same medium-to-long-length sample content and to strictly follow the tool prompts and parameters, thereby generating a complete set of item-level responses for each test. Each test was initiated in a separate, newly opened chat, resulting in 10 fully independent testing datasets. All test runs were conducted under fully blind-scoring, untuned, zero-shot prompt-based conditions, meaning the models received no prior contextual learning, no exposure to benchmark answers, no human-in-the-loop feedback, and relied exclusively on the instructions embedded in the Diet-MisRAT tool.

While the use of generative LLMs like ChatGPT for instrument validation remains relatively new^22,76^, recent developments in in-context learning (ICL) techniques such as zero-shot and few-shot prompting have opened new opportunities for their application in tasks like fake news detection^95,96^. OpenAI has not publicly disclosed the exact architecture of its models, although they are described as loosely mimicking certain aspects of human reasoning through billions of interconnected nodes (often referred to as ‘artificial neurons’), forming complex associations between words, concepts, and contextual patterns^97^. These capabilities make user-friendly LLMs useful for evaluating and refining a tool’s performance.

In this study, we used GenAI-based testing as a final, complementary layer to the professional human rounds, not only to help accelerate the testing process but also to examine the model’s performance, speed, and scalability in automatically applying the tool under zero-shot prompting conditions. This phase further allowed us to explore how widely accessible AI systems like ChatGPT might be able to support real-world misinformation risk management with human oversight^98^, especially in light of their fallible or insufficient ability to do so autonomously^74,99–101^.

### Scoring blindness across rounds

In all five rounds of Diet-MisRAT testing, human participants and ChatGPT completed the tool assessments independently and without access to the underlying weighting framework associated with each response option, the total possible scores, or the final risk characterisation (very low, low, moderate, high, or very high). The purpose of this design was to ensure that respondents relied exclusively on their interpretation of item prompts and risk-related cues, free from influence by the tool’s weighting structure or final scoring, thereby preventing any attempt to reverse-engineer a desired result or ‘game the system’. This set-up also allowed for a more objective evaluation of how clearly the items were understood and how consistently the tool could be applied across independent users and ChatGPT models.

### Tool application speed

Only ChatGPT model o3 displayed a system-generated speed indicator, allowing reliable capture of task durations, which averaged 25 s per medium-to-long-length content analysis. While this represents a speed over 90 times faster than the 37 min, 36 s averaged by highly experienced nutrition professionals, such duration may still prove limiting in high-volume scenarios if analyses are conducted sequentially. That said, with appropriate automated AI deployment, such as parallelised API solutions or batch-processing^102^, the tool could feasibly be scaled for simultaneous multi-piece analysis in future.

### Statistical analysis

Statistical analyses and visualisations were performed using Microsoft^®^ Excel^®^ for Microsoft 365 MSO (Version 2503, Build 16.0.18623.20116, 64-bit), the open-access statistical program Social Science Statistics (Version 2018), Zoho Survey’s built-in statistical analysis features, and Zoho Analytics (Zoho Corporation Pvt. Ltd.). Round 5 testing was conducted using ChatGPT under an enterprise-level ChatGPT Team License within a private login-protected workspace environment (OpenAI, L.L.C.).

Analyses considered both individual item scores and the resulting total scores which contributed to overall misinformation risk classification as very low (0.0–20.9%), low (21.0–40.9%), moderate (41.0–60.9%), high (61.0–80.9%), or very high (81.0–100%). A granular five-band result classification matrix was preferred over a basic binary system, as it offered greater sensitivity to score variation and allowed for finer discernment between levels of risk^69^.

To assess how closely participants in Rounds 2 to 4 aligned with the a priori benchmark answers established in Round 1, we calculated Pearson’s correlation coefficients (*r*) between each participant’s item-level scores and the corresponding benchmark values, serving as an indicator of concurrent criterion validity. Interpretation of *r* values followed established guidelines: coefficients between 0.00 and 0.10 were considered negligible, 0.10–0.39 weak, 0.40–0.69 moderate, 0.70–0.89 strong, and 0.90–1.00 very strong correlations^103^. For human participant rounds, we did not conduct a test-retest evaluation (i.e. repeated administration with the same raters over time), so the temporal stability of Diet-MisRAT scoring for individual users remains to be established in future work.

We noted and treated skipped responses in Rounds 2 to 4 as indicators of potential comprehension or interpretation challenges. Skipped responses were recorded and, where applicable, adjusted for in the analysis to ensure that incomplete data did not distort correlation outcomes. Participant feedback was also reviewed alongside these patterns and incorporated to inform refinements to the tool’s structure, content, and guidance.

We evaluated the internal consistency of the Diet-MisRAT as applied by the Round 4 cohort using Cronbach’s alpha, calculated across all items based on independently assigned responses. In line with established guidance, we interpreted this statistic as a conservative lower-bound estimate of reliability reflecting the minimum expected consistency, given that alpha coefficients may underestimate consistency when response values are non-equidistant or when multidimensionality is present^65,66^. Because Rounds 2 and 3 (postgraduate dietitians-in-training and postgraduate nutrition students) were designed primarily as interpretability and refinement rounds and showed higher item skipping and interpretive variability, we limited alpha estimation to the highly experienced nutrition professional cohort (Round 4), where responses were most complete and stable. Consequently, reliability evidence may not generalise to less experienced users, and additional reliability indices (e.g. split-half, inter-rater, and test–retest reliability) were not assessed, leaving reproducibility across cohorts and time points to be established.

We used Pearson’s correlation coefficients (*r*) in Round 5 to examine both concurrent criterion validity and test–retest reliability by comparing repeated applications of the tool by each ChatGPT model version (4o and o3), treating this as a supplementary check rather than a replacement for human reliability testing. Additionally, we compared model consistency and performance both between the two ChatGPT versions and against earlier human testing rounds, using median correlation values and statistical significance.

In Round 5, we also applied standard performance metrics commonly used in natural language processing (NLP) model evaluations and algorithmic content classification tasks, namely: accuracy, precision, sensitivity (also referred to as ‘recall’), and F1 scores^21,37,104–109^. Each Diet-MisRAT item presented a set of mutually exclusive categorical response options, mapped to preassigned but undisclosed (blinded) misinformation risk weights, and required the model to select a single forced-choice answer. Because the items were not structured as binary yes/no or true/false labels, we recalibrated model performance comparisons based on response alignment to expert benchmark responses and their pre-set risk weights. This recalibration enabled the quantification of two potential model misjudgement tendencies: risk overestimation (overflagging) and risk underestimation (underflagging).

Accuracy was calculated as the proportion of exact matches over total items, reflecting the overall percentage of fully benchmark-aligned responses selected by either model autonomously, solely relying on tool prompts, item guidance, and response options, without any prior training dataset or access to expert benchmark responses. Precision was defined as the proportion of exact matches among all exact-match responses and overflagged responses, gauging the model’s ability to avoid overflagging or overestimating risk. Sensitivity represented the proportion of exact matches among all exact-match responses and underflagged responses, measuring how well the model captured benchmark-flagged risks without underestimation. F1 score was computed as the harmonic mean of precision and sensitivity, penalising both risk overflagging and underflagging in a single metric. Performance formulas can be found in Supplementary Table S5.

All statistical comparisons were conducted using two-tailed tests, applying a significance threshold of *p* < 0.01.

## Supplementary Information

Below is the link to the electronic supplementary material.

Supplementary Material 1

## Data Availability

The data that support the findings of this study are available upon reasonable request from the corresponding author, A.R. The tool is stored at https://misinformation.science/, with access and use subject to licence restrictions.
