# Supplementary material for: Development and validation of a tool for detecting misinformation risk in diet, nutrition, and health content (Diet-MisRAT)
Source: Sci Rep. 2026 Mar 27;16:9207. doi: 10.1038/s41598-026-40534-2 (PMC13031550; doi:10.1038/s41598-026-40534-2)
Supplement: Supplementary file 1 — Supplementary Material 1 [file 41598_2026_40534_MOESM1_ESM.docx]

**Development and Validation of a Tool for Detecting Misinformation Risk in Diet, Nutrition, and Health Content
(Diet-MisRAT)**

Supplementary Information

**Alex Ruani^1,2,*^, Michael J Reiss^1^, Anastasia Z Kalea^3,4^**

**^1^** Curriculum, Pedagogy and Assessment, Institute of Education, University College London, London WC1H 0AL, UK

**^2^** The Health Sciences Academy, London SW6 5UA, UK

**^3^** Faculty of Medical Sciences, Division of Medicine, University College London, London WC1E 6BT, UK

*To whom correspondence may be addressed. Email: [maria.ruani.17@ucl.ac.uk](mailto:maria.ruani.17@ucl.ac.uk)

**Keywords:** Misinformation Risk Assessment

**Supplementary Figures**

**Supplementary Figure S1. The Four Core Risk Dimensions Contributing to Diet-Health Misinformation**


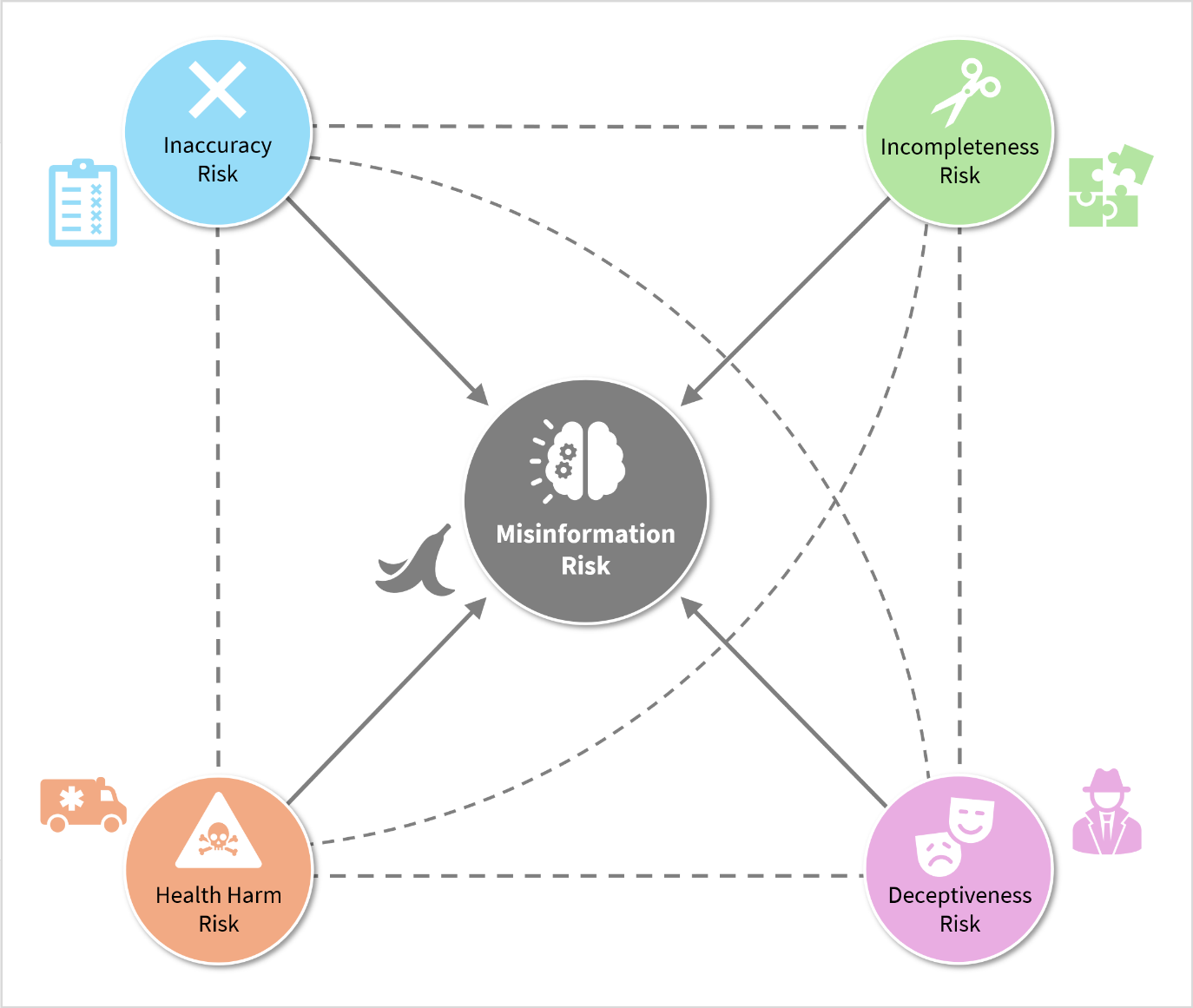


Diagram illustrating the four core misinformation risk dimensions in diet-health content: 1) inaccuracy, 2) incompleteness, 3) deceptiveness, and 4) health harm, all of which contribute to overall misinformation risk (solid lines). The four dimensions are also interconnected with one another (dotted lines), indicating mutual influence and shared underlying traits. Each risk dimension can qualitatively and quantitatively overlap, meaning multiple dimensions may co-occur within a single piece of content, thereby compounding and amplifying the total misinformation risk. See **Supplementary Table S1** for a brief conceptualisation of each dimension and examples of representative misinformation risk factors.

**Supplementary Figure S2. Round 1 concurrent validity, item-level response trends, and risk classification via the Diet-MisRAT**


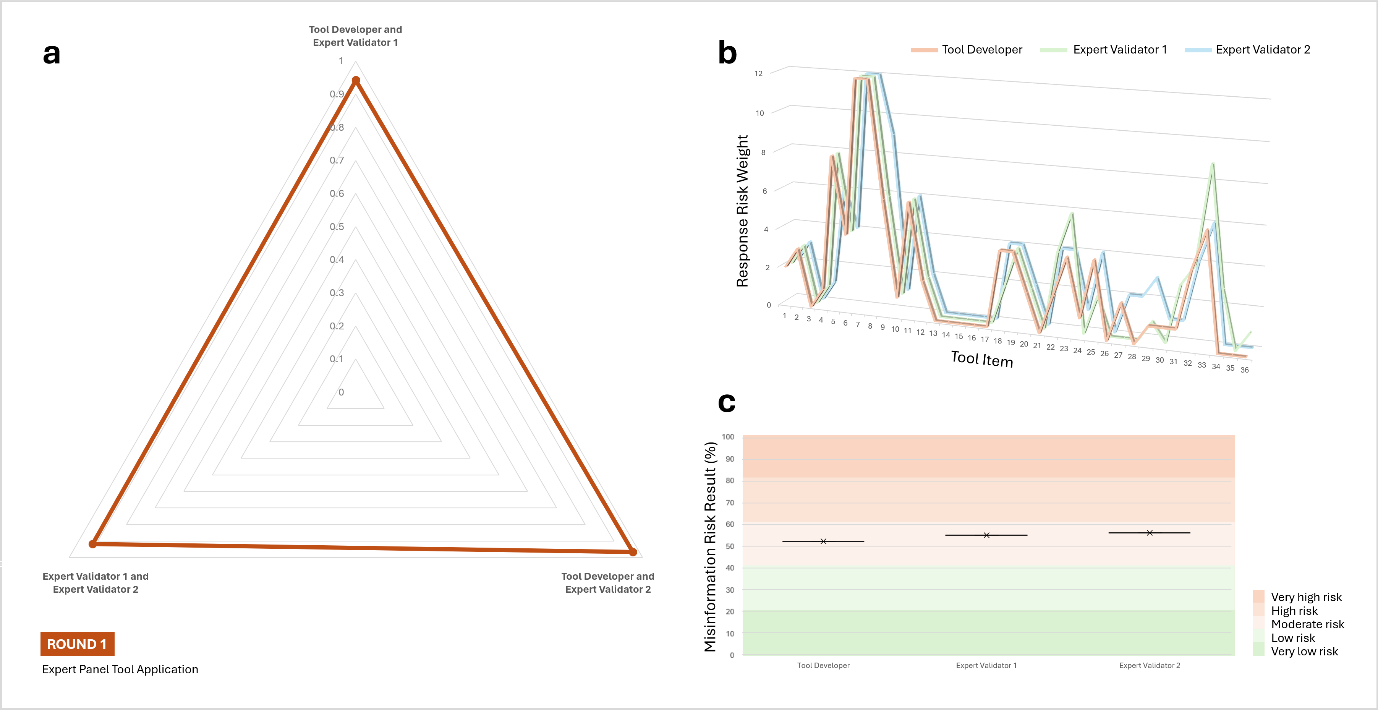


**a.** Radar plot showing the strength of association between tool developer and expert validators’ total item responses, as measured by Pearson’s correlation coefficient (*r*). All two-tailed comparisons were statistically significant (*p* < 0.00001). **b.** Line graph comparing item-level scoring trends (response risk weights) across all tool items. Each line represents an expert user’s scoring pattern. **c.** Risk band plot with stratified background, displaying misinformation risk outcomes derived from each expert user’s total tool score (black cross), expressed as a percentage of the maximum possible score after recalibration to a 100% scale. Background shading indicates five misinformation risk bands: very low (0–20.9%), low (21.0–40.9%), moderate (41.0–60.9%), high (61.0–80.9%), and very high (81.0–100%). Sample size: *N* = 3 (Round 1 expert panel).

*Note*: Expert validator 2, who has an extensive background in nutrition science research and education, demonstrated higher agreement with the tool developer, closely reflecting the tool’s intended interpretation. The correlation between the two expert validators was also very strong (*r* = 0.92, *p* < 0.00001), though slightly lower than their individual correlations with the developer. This suggests both experts were more closely aligned with the tool developer’s scoring rationale than with each other (see **Supplementary Fig. S2**, panels a–c).

**Supplementary Tables**

**Supplementary Table S1. The Four Core Risk Dimensions Contributing to Diet-Health Misinformation**

©Copyright, Alex Ruani, 2025. All Rights Reserved.

| Risk Dimension | What It Captures | Misinformation Risk Impact | Representative Risk Factors  (Illustrative, Non-exhaustive) |
| --- | --- | --- | --- |
| Inaccuracy  Risk | Information that is illogical, incoherent, defective, misrepresented, or lacks alignment with current scientific constructs. | Alters perception of diet-health concepts or distorts understanding, whether intentionally or not. | - Internal contradictions or inconsistencies - Overgeneralisations - Causation or reversal claims from correlation - Technical clickbait (headline/content mismatch) - Misrepresented evidence (e.g. false balance or equivalence, cherry-picking, framing relative risk as absolute risk) - Exaggerated or decontextualised claims (e.g. overextended health promises, virtue-washing, health halo) - Presenting heuristic or intuitive reasoning as evidence (e.g. fallacious logic, misapplied plausibility) - Reliance on subpar or dubious sources - Denial of established scientific consensus |
| Incompleteness Risk | Information that lacks critical details, context, or full disclosure. | Can mislead by omission, even when technically appropriate or aligned with current evidence. | - Missing crucial contextual details - Omission of caveats, limitations, or exceptions - Neglected risks or safety precautions - Omission of additional paths to the same goal (e.g. misrepresenting one path as the only option) - Exclusion of substantiated alternative viewpoints - Lack of specification about subgroups or applicable contexts - Unverifiable references - Absence of peer review - Omission of existing uncertainty |
| Deceptiveness Risk | Information that conceals authorship, intent, source reliability, affiliations, or uses deceptive or manipulative framing that distorts perception.  Strongly associated with greater **disinformation risk** (i.e. intentionality to mislead). | Signals possible intent to mislead, manipulate, or deceive, even when content contains scientific language or partial evidence. | - Undisclosed conflicts of interest - Fake or borrowed credibility - Vague or falsified credentials - Expert mimicry or impersonation - Reliance on deepfakes or shallow fakes - Selective disclosure - Artificial transparency - Trust-building features without verifiable sources - Illusion of evidence or certainty - False-hope appeals (e.g. promises of cures or guaranteed efficacy/safety) - Emotional manipulation tones or techniques (e.g. fear-mongering, conspirative, polarising, outrage baiting, overly optimistic or uncritical, satirical) |
| Health Harm Risk | Information that increases the likelihood of health or dietary decisions leading to harm or posing a risk to life.  At times associated with **malinformation risk** (e.g. where accurate information is presented or deployed in ways that may cause harm). | Carries the gravest (mis)informational adverse effects, whether intentionally or inadvertently. | - Overstating benefits while downplaying risks - Promoting unsafe practices or behavioural changes - Omitting contraindications or safety precautions - Exaggerating safety claims or risk warnings   ©Copyright, Alex Ruani, 2025. All Rights Reserved.   - Suggesting diet as a replacement of essential medical care - Encouraging rejection of life-saving public health guidance - Misleading supplement recommendations (e.g. omission of safe intake limits or toxicity thresholds, failure to account for dangerous food–drug interactions) - Promising ‘cures’ or complete safety - Rejecting established scientific consensus or public guidance in ways that may endanger health or life itself |

*Note*: This table outlines the four distinct but interrelated risk dimensions assessed by the Diet-MisRAT tool: 1) inaccuracy, 2) incompleteness, 3) deceptiveness, and 4) health harm. Each dimension captures a unique aspect of misinformation risk, ranging from factual distortions and risky omissions to concealed intent and potentially harmful consequences. These dimensions were derived to support systematic detection and classification of misinformation risk factors in diet-health content, enabling more granular and actionable risk assessment. Some risk factors may fall under more than one dimension, compounding their potential to mislead or cause harm. The examples provided are illustrative and non-exhaustive. See **Supplementary Fig. S1** for a visual representation of these four risk dimensions.

**Supplementary Table S2. Integrated Misinformation Risk Categorisation and Response Guidance**

©Copyright, Alex Ruani, 2025. All Rights Reserved.

| Misinformation Risk Level | Risk  Description | Potential Impact on Recipients | Risk Management and Risk Communication Recommendations |
| --- | --- | --- | --- |
| High Risk or Very High  Risk | There is a **high** or **very high likelihood** that a recipient (or group of recipients) could become misinformed or be misled by the assessed content, **likely** increasing the risk of harmful outcomes (e.g. detrimental impact on psychological or physical health, survival, or life itself) based on the **severity** and **combined influence** of misinformation risk factors present in the content. | The assessment identified **numerous or severe** specific risk factors in the content of interest, indicating a **high likelihood** of misinformation occurring – i.e. **highly likely** to be present or to be encountered by the recipient/s, increasing their susceptibility to:   - accept the content (or aspects of it) to varying degrees of misperceived trustworthiness, reliability, safety, credibility, coherence, completeness, or other relevant measures, - become misinformed or misled by it (e.g. through the shaping/reinforcement of misinformed beliefs, or misdirected attitudinal orientation), or - engage in misguided decision-making based on it (e.g. adopting harmful behaviours or forgoing life-saving ones, publicly amplifying or spreading risky content, intending to act or abstain based on inaccurate, unsafe, or incomplete understanding), among other **highly likely** informational adverse effects. | For content assessed as **high** or **very high risk**, risk managers and communicators (including AI-supported systems) may consider the following sequential actions:  1) **Flag** the content in back-end systems as ‘high-risk’ or ‘very-high-risk’, and **list** the salient risk factors, prioritising those related to the probability of health harm. *NOTE:* These systems may not only include user-to-user and search services (such as social media platforms, search engines, and generative AI interfaces), but also novel individual user installations (such as emerging web-browser add-ons/extensions or mobile apps that users can opt into, designed to flag and block risky content in a user’s own devices).  2) **Neutralise** high-harm-risk content and its duplicates where feasible (e.g. through takedowns where harm-risk indicators are exceedingly high, algorithmic penalties or demotion, down-listing or de-indexing from search results, or hiding from user view – much like how user-installed ad-blockers filter digital clutter before exposure).  4) **Tag** or index the content as ‘high-risk’ or ‘very-high-risk' in a shared central database or quarantine repository to support cross-platform flagging and to enable detection by multiple systems through content matching, reducing the need for repeated misinformation risk assessments on the same content.  4) **Monitor** the content originators and active spreaders to identify patterns of similar content flooding or other high-risk content dissemination.  5) Where content cannot be removed or blocked by a system, **forewarn** recipients proactively, right before access or full exposure. For example:  (a) In search results, **display** a red-flag or traffic-light warning before full access (e.g. *“This content may be misleading and unsafe”;* akin to traffic light warnings on food packaging before consumption).  (b) In web-browser applications or mobile installations, **trigger** alerts, risk prompts, and gamified features for interactive inoculation embedded in the user experience (UX) before full exposure to the risky content, including incentives for sharing the preventative inoculation messaging with other users or groups).  (c) **Introduce** sharing friction cues that appeal to moral or social values, such as popup alters or nudges before proceeding to share the risky content (e.g. *“Are you sure you want to share this? It may mislead others in ways that lead to harm”*).  ©Copyright, Alex Ruani, 2025. All Rights Reserved.  6) Where the high-risk content has spread widely or there is a high probability of reaching and impacting many recipients, **develop** and deploy rapid counter-messaging, such as targeted pre-bunks (e.g. narrow spectrum, issue-based) and debunks (corrections) for public-facing health communication and education. This is especially critical during public health emergencies and disruptive infodemic incidents, to help mitigate harm.  7) Before content is published, content-creation applications could issue a **caution** that the material has a high risk of misinforming recipients in ways that may lead to harm, **prompting authors or editors** to review the flagged issues before publishing (e.g. through underlined text or a checklist of identified risks that need addressing), including guidance on how to reduce this overall risk by tackling each individual risk factor (functioning like a ‘*Grammarly’* of misinformation-risk reduction within the author’s own content). This could help improve the quality of content creators’ materials. |
| Moderate  Risk  ©Copyright, Alex Ruani, 2025. All Rights Reserved. | There is a **moderate likelihood** that a recipient (or group of recipients) could become misinformed or be misled by the assessed content, **potentially** increasing the risk of harmful outcomes (e.g. detrimental impact on psychological or physical health, survival, or life itself) based on the **nature** and **combined influence** of misinformation risk factors present in the content.  ©Copyright, Alex Ruani, 2025. All Rights Reserved. | The assessment identified **several or material** specific risk factors in the content of interest, indicating a **moderate likelihood** of misinformation occurring – i.e. **moderately likely** to be present or to be encountered by the recipient/s, increasing their susceptibility to:   - accept the content (or aspects of it) to varying degrees of misperceived trustworthiness, reliability, safety, credibility, coherence, completeness, or other relevant measures, - become misinformed or misled by it (e.g. through the shaping/reinforcement of misinformed beliefs, or misdirected attitudinal orientation), or - engage in misguided decision-making based on it (e.g. adopting harmful behaviours or forgoing life-saving ones, publicly amplifying or spreading risky content, intending to act or abstain based on inaccurate, unsafe, or incomplete understanding), among other **potential** informational adverse effects. | Risk communicators and managers should not rely solely on the overall ‘**moderate**’ misinformation risk label, but also incorporate health harm risk indicators as an essential dimension in their response strategy.  **Considerations for Moderate Misinformation Risk with Elevated Health Harm Potential:**  Even when the overall content is categorised as posing only a moderate risk of misinformation, the detailed risk assessment may still reveal a high probability of health harm. This indicates that, despite a moderate likelihood of misinformation presence overall, the consequences for psychological or physical health/wellbeing, survival, or life itself may still be severe if recipients act on the content.  In these instances, it is advisable to apply precautionary mitigation strategies akin to those recommended for high-risk content, such as:  1) **Neutralising,** demoting, or removing the content when the risk of harm it poses is immediate, extensive, or irreversible.  2) **Implementing** targeted warnings, alerts, or friction prompts to forewarn users prior to exposure (e.g. through what we call ‘gamified UX inoculation’) as well as risk nudges before sharing.  3) **Flagging** the content and its specific risk indicators in back-end systems and centralised databases or quarantine repositories to enable detection across platforms.  4) **Developing** and disseminating public health counter-messaging, such as tailored pre-bunks or debunks, especially in crisis scenarios where rapid responses may help reduce health harm risk. |
| Low Risk or  Very Low  Risk | There is a **low** or **very low likelihood** that a recipient (or group of recipients) would become misinformed or be misled by the assessed content in a way that contributes to harmful outcomes. While the possibility of health- or life-related harm risk cannot be entirely ruled out if recipients act on the content, the probability of this is considered to be **low** or **very low** based on the nature and extent of other risk factors assessed in the content.  ©Copyright, Alex Ruani, 2025. All Rights Reserved. | The assessment identified **few or only minor** specific risk factors in the content of interest, indicating a **low** or **very low likelihood** of misinformation occurring – i.e. **unlikely** to be present or to significantly influence the recipient/s, with a **lower probability** that they would:   - accept the content (or aspects of it) to varying degrees of misperceived trustworthiness, reliability, safety, credibility, coherence, completeness, or other relevant measures, - become misinformed or misled by it (e.g. through the shaping/reinforcement of misinformed beliefs, or misdirected attitudinal orientation), or - engage in misguided decision-making based on it (e.g. adopting harmful behaviours or forgoing life-saving ones, publicly amplifying or spreading risky content, intending to act or abstain based on inaccurate, unsafe, or incomplete understanding), among other potential but **less likely** informational adverse effects. | Assigning a **low** or **very low risk** status may position the content as preferable for distribution and user engagement, potentially granting it greater visibility over higher-risk materials and ultimately helping to recalibrate current platform incentive structures.  To promote a culture of responsible information flow while maintaining vigilance, the following actions may be considered by risk managers and communicators:  1) **Display** a positive tag (e.g. tick, star, ‘trusted’ labels, or other reassuring visual cue) to incentivise healthier content consumption, engagement, and sharing. This would not only function as a green light for users, but also support a cumulative ‘gold star’ designation for platforms, potentially incentivising their promotion of healthier content.  2) **Encourage** positive amplification of safe, high-quality content (e.g. promote in ‘for you’ and topic feeds), prioritise as part of feed or search results diversification to sustain exposure balance, assign higher ranking eligibility for search AI summaries, include in ‘best answer’ or ‘top sources’ panels, minimise consumption or sharing friction, and classify for enclosure in educational hubs, grounding collections, or risk-attenuating counter-speech repositories  3) **Apply** age-gating or other audience-safe delivery where relevant, and allow a professional review, even when content is considered relatively low risk, to ensure that:   - no severe risk factor has been overlooked or underweighted (in particular, health harm risk indicators), - opportunities for preventive inoculation (e.g. misleading framings or other risk factors) are identified and flagged for educational purposes, and - content remains appropriate for diverse audiences with varying literacy or vulnerability levels.   3) **Monitor** for content or contextual evolution, particularly where originally low-risk material may become problematic over time due to:   - public reinterpretation, distortive reframing, misattribution, or viral misuse, - shifts in scientific consensus or public health guidance, or - emergence of high-quality, widely accepted evidence that corrects or recontextualises earlier claims.   4) **Reassess** the risk if the same content is repurposed or reframed in a new format (such as a decontextualised quote on social media, an AI-generated summary, or a meme), as the context may alter its impact or likelihood of misinforming.  ©Copyright, Alex Ruani, 2025. All Rights Reserved.  5) **Index** as ‘low risk’ or ‘very low risk’ in shared or centralised databases to support triaging and clear differentiation from moderate or higher risk content, reducing unnecessary repeat assessments. |

*Note*: This table provides a structured framework for interpreting overall misinformation risk results from Diet-MisRAT assessments, stratified into five levels (very low, low, moderate, high, and very high risk). For each risk category, it outlines: a) a brief description of the assessed misinformation risk level, b) the potential impact on recipients (e.g. susceptibility to misinformed beliefs or harmful behaviours), and c) illustrative response strategies for content moderation, public health communication, and risk mitigation. It is intended to support decision-making by researchers, risk communicators, ethicists, digital platforms, AI systems, and public health bodies in managing misinformation exposure. This includes determining when to flag, suppress, neutralise, monitor, or counter-message risky content, based on both the likelihood of misinformation and the potential for health-related harm. The table also highlights when moderate-risk content may warrant high-risk responses due to elevated harm potential, and offers practical actions to triage or pre-empt future risk escalation.

The stratification of content risk severity is consistent with regulatory expectations outlined by Ofcom for online safety and risk management in digital platforms and search services, particularly those described in its risk assessment guidance for illegal and harmful content[1].

References:

**1.** Ofcom. *Protecting people from illegal harms online: Risk assessment guidance and risk profiles.* <https://www.ofcom.org.uk/siteassets/resources/documents/online-safety/information-for-industry/illegal-harms/risk-assessment-guidance-and-risk-profiles.pdf?v=390984> (December 2024).

**Supplementary Table S3. Summary of Testing Rounds 1 to 4 Results**

**Round 1 Expert Panel Validation**

| **Expert Users** | **Pearson’s *r**** | **Correlation** | ***p*-value** | **Misinformation Risk Level (%)** | **Misinformation Risk Category**** |
| --- | --- | --- | --- | --- | --- |
| Expert Validator 1 | 0.94 | Very Strong | < 0.00001 | 55.1 | Moderate |
| Expert Validator 2 | 0.97 | Very strong | < 0.00001 | 56.2 | Moderate |
| Expert Validator 1 vs Expert Validator 2 | 0.92 | Very strong | < 0.00001 | -- | |
| Tool Developer | -- | | | 52.2 | Moderate |
| Expert Benchmark | -- | | | 53.4 | Moderate |

*Pearson’s *r* reflects the item-level correlation between each expert validator’s responses and those of the tool developer, as well as between the two validators.

**Misinformation risk bands: very low (0–20.9%), low (21.0–40.9%), moderate (41.0–60.9%), high (61.0–80.9%), and very high (81.0–100%).

**Round 2 Testing (*N* = 7) with Postgraduate Dietitians in Training**

| **Participant** | **Pearson’s *r**** | **Correlation** | ***p*-value** | **Misinformation Risk Level (%)** | **Misinformation Risk Category**** |
| --- | --- | --- | --- | --- | --- |
| P1 | 0.81 | Strong | < 0.00001 | 60.7 | Moderate |
| P2 | 0.94 | Very strong | < 0.00001 | 50.6 | Moderate |
| P3 | 0.68 | Moderate | < 0.00001 | 38.2 | Low |
| P4 | 0.92 | Very strong | < 0.00001 | 47.8 | Moderate |
| P5 | 0.85 | Strong | < 0.00001 | 63.5 | High |
| P6 | 0.86 | Strong | < 0.00001 | 46.6 | Moderate |
| P7 | 0.92 | Very strong | < 0.00001 | 57.9 | Moderate |
| **Expert Benchmark** | -- | | | 53.4 | Moderate |
| **Median Pearson’s *r*** | 0.86 | Strong | -- | | |

*Pearson’s *r* represents the correlation between each participant’s item-level responses (P1 to P7) and the expert-derived benchmark responses.

**Misinformation risk bands: very low (0–20.9%), low (21.0–40.9%), moderate (41.0–60.9%), high (61.0–80.9%), and very high (81.0–100%).

**Round 3 Testing (*N* = 33) with Postgraduate Nutrition Students**

| **Participant** | **Pearson’s *r**** | **Correlation** | ***p*-value** | **Misinformation Risk Level (%)** | **Misinformation Risk Category**** |
| --- | --- | --- | --- | --- | --- |
| P1 | 0.60 | Moderate | 0.00011 | 36 | Low |
| P2 | 0.78 | Strong | < 0.00001 | 37.6 | Low |
| P3 | 0.93 | Very Strong | < 0.00001 | 43.3 | Moderate |
| P4 | 0.69 | Moderate | < 0.00001 | 43.3 | Moderate |
| P5 | 0.94 | Very Strong | < 0.00001 | 45.5 | Moderate |
| P6 | 0.84 | Strong | < 0.00001 | 46.6 | Moderate |
| P7 | 0.41 | Moderate | 0.012747 | 47.8 | Moderate |
| P8 | 0.91 | Very Strong | < 0.00001 | 47.8 | Moderate |
| P9 | 0.92 | Very Strong | < 0.00001 | 49.4 | Moderate |
| P10 | 0.83 | Strong | < 0.00001 | 52.2 | Moderate |
| P11 | 0.91 | Very Strong | < 0.00001 | 52.8 | Moderate |
| P12 | 0.74 | Strong | < 0.00001 | 53.9 | Moderate |
| P13 | 0.67 | Moderate | 0.000012 | 54.5 | Moderate |
| P14 | 0.90 | Very Strong | < 0.00001 | 55.1 | Moderate |
| P15 | 0.75 | Strong | < 0.00001 | 56.2 | Moderate |
| P16 | 0.62 | Moderate | 0.000065 | 58.4 | Moderate |
| P17 | 0.89 | Strong | < 0.00001 | 60.7 | Moderate |
| P18 | 0.84 | Strong | < 0.00001 | 60.7 | Moderate |
| P19 | 0.88 | Strong | < 0.00001 | 60.7 | Moderate |
| P20 | 0.91 | Very Strong | < 0.00001 | 61.2 | High |
| P21 | 0.66 | Moderate | 0.000011 | 68 | High |
| P22 | 0.76 | Strong | < 0.00001 | 71.9 | High |
| P23 | 0.57 | Moderate | 0.000343 | 36.5 | Low |
| P24 | 0.79 | Strong | < 0.00001 | 38.2 | Low |
| P25 | 0.91 | Very Strong | < 0.00001 | 42.1 | Moderate |
| P26 | 0.94 | Very Strong | < 0.00001 | 45.5 | Moderate |
| P27 | 0.84 | Strong | < 0.00001 | 46.6 | Moderate |
| P28 | 0.76 | Strong | < 0.00001 | 48.3 | Moderate |
| P29 | 0.81 | Strong | < 0.00001 | 51.1 | Moderate |
| P30 | 0.73 | Strong | < 0.00001 | 51.1 | Moderate |
| P31 | 0.88 | Strong | < 0.00001 | 56.2 | Moderate |
| P32 | 0.88 | Strong | < 0.00001 | 56.7 | Moderate |
| P33 | 0.81 | Strong | < 0.00001 | 64.6 | High |
| **Expert Benchmark** | -- | | | 53.4 | Moderate |
| **Median Pearson’s *r*** | 0.83 | Strong | -- | | |

*Pearson’s *r* represents the correlation between each participant’s item-level responses (P1 to P33) and the expert-derived benchmark responses.

**Misinformation risk bands: very low (0–20.9%), low (21.0–40.9%), moderate (41.0–60.9%), high (61.0–80.9%), and very high (81.0–100%).

**Round 4 Testing (*N* = 15) with Highly Experienced Nutrition Professionals**

| **Participant** | **Pearson’s *r**** | **Correlation** | ***p*-value** | **Misinformation Risk Level (%)** | **Misinformation Risk Category**** |
| --- | --- | --- | --- | --- | --- |
| P1 | 0.88 | Strong | < 0.00001 | 42.1 | Moderate |
| P2 | 0.94 | Very strong | < 0.00001 | 62.4 | High |
| P3 | 0.93 | Very strong | < 0.00001 | 52.2 | Moderate |
| P4 | 0.92 | Very strong | < 0.00001 | 63.5 | High |
| P5 | 0.93 | Very strong | < 0.00001 | 60.7 | Moderate |
| P6 | 0.83 | Strong | < 0.00001 | 70.2 | High |
| P7 | 0.89 | Strong | < 0.00001 | 61.2 | High |
| P8 | 0.93 | Very strong | < 0.00001 | 57.3 | Moderate |
| P9 | 0.82 | Strong | < 0.00001 | 44.4 | Moderate |
| P10 | 0.97 | Very strong | < 0.00001 | 53.4 | Moderate |
| P11 | 0.95 | Very strong | < 0.00001 | 53.9 | Moderate |
| P12 | 0.78 | Strong | < 0.00001 | 66.3 | High |
| P13 | 0.97 | Very strong | < 0.00001 | 52.2 | Moderate |
| P14 | 0.89 | Strong | < 0.00001 | 49.4 | Moderate |
| P15 | 0.90 | Very Strong | < 0.00001 | 49.4 | Moderate |
| **Expert Benchmark** | -- | | | 53.4 | Moderate |
| **Median Pearson’s *r*** | 0.92 | Very strong | -- | | |
| **Cronbach’s Alpha** | 0.73 | -- | | | |

*Pearson’s *r* represents the correlation between each participant’s item-level responses (P1 to P15) and the expert-derived benchmark responses.

**Misinformation risk bands: very low (0–20.9%), low (21.0–40.9%), moderate (41.0–60.9%), high (61.0–80.9%), and very high (81.0–100%).

**Supplementary Table S4. Summary of Performance Metrics Across ChatGPT Test Runs Guided by Diet-MisRAT Prompts**

| Model Group | Test Run | Pearson’s *r** | Correlation Strength | *p*-value | Accuracy (%) | Precision (%) | Sensitivity (%) | F1** Score (%) |
| --- | --- | --- | --- | --- | --- | --- | --- | --- |
| ChatGPT 4o | Test 1 | 0.98 | Very strong | <0.00001 | 94.4 | 97.1 | 94.4 | 95.8 |
|  | Test 2 | 0.99 | Very strong | <0.00001 | 94.4 | 97.1 | 94.4 | 95.8 |
|  | Test 3 | 0.99 | Very strong | <0.00001 | 91.7 | 97.1 | 91.7 | 94.3 |
|  | Test 4 | 0.99 | Very strong | <0.00001 | 94.4 | 97.1 | 94.4 | 95.8 |
|  | Test 5 | 0.99 | Very strong | <0.00001 | 94.4 | 97.1 | 94.4 | 95.8 |
|  | **Median** | 0.99 | Very strong | - | 94.4 | 97.1 | 94.4 | 95.8 |
|  | **Mean  ± SD** | - | - | - | 93.9  ± 1.2 | 97.1  ± 0.0 | 93.9  ± 1.2 | 95.5  ± 0.7 |
|  | **95% CI** [LL, UL] | - | - | - | [90.4, 97.4] | [94.7, 99.6] | [90.4, 97.4] | [92.4, 98.5] |
| ChatGPT o3 | Test 1 | 0.97 | Very strong | <0.00001 | 86.1 | 96.9 | 86.1 | 91.2 |
|  | Test 2 | 0.96 | Very strong | <0.00001 | 83.3 | 100.0 | 83.3 | 90.9 |
|  | Test 3 | 0.97 | Very strong | <0.00001 | 83.3 | 100.0 | 83.3 | 90.9 |
|  | Test 4 | 0.98 | Very strong | <0.00001 | 83.3 | 96.8 | 83.3 | 89.6 |
|  | Test 5 | 0.96 | Very strong | <0.00001 | 86.1 | 96.9 | 86.1 | 91.2 |
|  | **Median** | 0.97 | Very strong | - | 83.3 | 96.9 | 83.3 | 90.9 |
|  | **Mean  ± SD** | - | - | - | 84.4  ± 1.5 | 98.1  ± 1.7 | 84.4  ± 1.5 | 90.7  ± 0.7 |
|  | **95% CI** [LL, UL] | - | - | - | [79.1, 89.7] | [96.1,  100] | [79.1, 89.7] | [86.5, 95.0] |
| Combined | **Median** | 0.98 | Very strong | - | 88.9 | 97.1 | 88.9 | 92.7 |
|  | **Mean ± SD** | - | - | - | 89.2  ± 5.1 | 97.6  ± 1.3 | 89.2  ± 5.1 | 93.1  ± 2.6 |
|  | **95% CI** [LL, UL] | - | - | - | [84.6, 93.7] | [95.4, 99.8] | [84.6, 93.7] | [89.4, 96.8] |

*Pearson’s *r* represents the correlation between each test run’s item-level responses and the expert-derived benchmark responses.

**F1 score refers to the effectiveness (‘F’) measure of performance, where precision and sensitivity are equally weighted (*β* = 1) and combined using the harmonic mean (see **Supplementary Table S5** for formula).

*Note:* Performance values are shown for both GenAI model versions (ChatGPT 4o and o3) across five independent test runs each, based on structured application of the Diet-MisRAT evaluation tool. Metrics include Pearson’s *r*, correlation strength, *P* values, and four standard performance measures: accuracy, precision, sensitivity (recall), and F1 score. For each model, median values, mean ± standard deviation (SD), and 95% confidence intervals (CIs) are reported. Confidence intervals were calculated using normal approximation for proportions (*N* = 180 per model group). Combined rows reflect pooled results across both models (*N* = 360). All *P* values reflect two-tailed Pearson correlation significance testing against expert benchmark responses.

**Supplementary Table S5. AI Performance Formulae**

| **Performance Metric** | **Formula** | **Description** |
| --- | --- | --- |
| **Accuracy** | $\frac{Exact Matches}{Total Items}$ | Reflects the overall proportion of benchmark-aligned responses selected by the AI model, solely relying on tool prompts in zero-shot, blinded-scoring conditions. |
| **Precision** | $\frac{Exact Matches}{Exact Matches + Overflagged Responses}$ | Reflects the AI model’s ability to avoid overflagging or overestimating risk. This formula penalises risk overflagging. |
| **Sensitivity (Recall)** | $\frac{Exact Matches}{Exact Matches + Underflagged Responses}$ | Indicates how well the AI model captured benchmark-flagged risks without underestimation. This formula penalises risk underflagging. |
| **F1 Score** | $\frac{2 x (Precision x Sensitivity)}{Precision + Sensitivity}$ | Balances precision and sensitivity, penalising both risk overflagging and risk underflagging by the AI model in a single metric. |

*Note:* This table contains the performance metric formulae (and descriptions) used to evaluate GenAI model alignment with expert benchmark responses under zero-shot prompting conditions. Response types: exact match, overflagged, underflagged. *Exact match:* AI model-selected response aligned precisely with the benchmark response. *Overflagged:* AI model-selected response carried a higher risk weight than the benchmark. *Underflagged:* AI model-selected response carried a lower risk weight than the benchmark.

**Appendix A. Participant Demographics**

**Round 2 Testing (*N* = 7) – Postgraduate Dietitians in Training**

| Variable | Category | *N* | % |
| --- | --- | --- | --- |
| Age | 21–25 | 5 | 71 |
|  | 26–30 | 1 | 14 |
|  | 31–35 | 1 | 14 |
| Gender | Female | 6 | 86 |
|  | Male | 1 | 14 |
|  | Other | 0 | 0 |
| English Proficiency | Native speaker | 6 | 86 |
|  | Fluent/proficient | 1 | 14 |

**Round 3 Testing (*N* = 33) – Postgraduate Nutrition Students**

| Variable | Category | *N* | % |
| --- | --- | --- | --- |
| Gender | Female | 28 | 85 |
|  | Male | 4 | 12 |
|  | Other | 1 | 3 |
| Age Group | 21–25 | 19 | 58 |
|  | 26–30 | 6 | 18 |
|  | 31–35 | 5 | 15 |
|  | 36–40 | 1 | 3 |
|  | 46–50 | 1 | 3 |
|  | 51–55 | 1 | 3 |
| English Proficiency | Native speaker | 12 | 36 |
|  | Fluent/proficient | 6 | 18 |
|  | Advanced | 4 | 12 |
|  | Very good | 1 | 3 |
|  | Good | 7 | 21 |
|  | Standard | 3 | 9 |
|  | **Total native or fluent** | 18 | 55 |
|  | **Lower proficiency (neither native nor fluent)** | 15 | 45 |

**Round 4 Testing (*N* = 15) – Highly Experienced Nutrition Professionals**

| Variable | Category | *N* | % |
| --- | --- | --- | --- |
| Years of Experience | 30 years or more | 2 | 13 |
|  | 20–29 years | 6 | 40 |
|  | 10–19 years | 5 | 33 |
|  | 8-9 years | 2 | 13 |
| Highest Academic Level | PhD | 9 | 60 |
|  | MSc | 4 | 27 |
|  | BSc | 2 | 13 |
| Gender | Female | 11 | 73 |
|  | Male | 4 | 27 |
|  | Other | 0 | 0 |
| Age Group | 30–45 years | 6 | 40 |
|  | 46–55 years | 5 | 33 |
|  | 56–65 years | 4 | 27 |
|  | **Median age range** | 46–55 years |  |
| English Proficiency | Native speaker | 10 | 67 |
|  | Fluent/proficient | 5 | 33 |

*Note*: Demographic information was collected within the same questionnaire that contained the assessment tool. The higher representation of females aligns with broader trends in the nutrition and healthcare sectors, where females often constitute the majority of the workforce[1].

References:

**1.** Health Careers. *Dietetics – career choices factsheet*. National Health Services (NHS) Health Careers. Available at: <https://test.aylesburyutc.co.uk/wp-content/uploads/2024/01/Dietetics-Career-Choices-factsheet.pdf> (Accessed 13 June 2025).

**Appendix B. ChatGPT Models at the Time of Round 5 Testing***


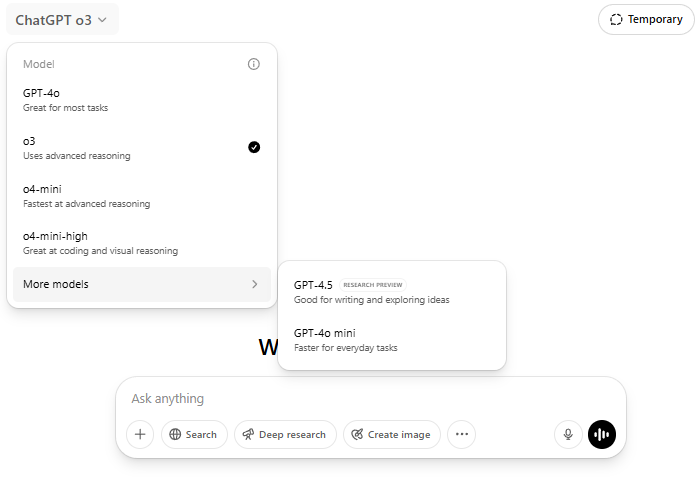


**Screenshot date: 3 May 2025 (https://chatgpt.com/).*

**Appendix C. General Tool Features**

The final iteration of the DietMisRAT instrument comprised a structured multiple-choice questionnaire designed to evaluate misinformation risk in lay content related to diet, nutrition, and health. The tool featured:

- 36 assessment items (each item framed as a content analysis prompt or question), organised into 19 categories.
- Guidance text and instructions accompanying each item.
- 137 pre-defined response options, which also acted as recognition prompts or cues to support the identification of risk features in content; these were qualitative, quantitative, or a combination of both.
- Pre-assigned weights per response option, contributing to the overall risk score, with items using three to seven response options depending on the construct assessed (i.e. response sets and scoring ranges were not interchangeable across items).
- A stratified multiplier system, applying coefficients depending on the intrinsic severity risk of each item – e.g. 1 (low risk), 2 (moderate risk), or 3 (high risk), where items directly associated with greater harm risk were proportionately assigned higher risk weights.
- A final five-level misinformation risk result: very low, low, moderate, high, and very high.

**Tool Prompting Structure and Specification**

Each item in the tool was framed as a content analysis prompt or question, designed to guide users and AI systems in evaluating the content of interest in a consistent and structured manner, without requiring extensive external verification. The prompts were constructed to focus the user’s (or system’s) attention on identifying specific risk factors within the content associated with the likelihood of misinforming recipients. Possible response options were qualitative, quantitative, or mixed, encouraging active engagement with the content and directing attention on distinct features requiring qualitative and quantitative evaluation.
